# Polymers Containing Diethylsiloxane Segment and Active Functional Group by Ring-Opening Polymerization of Hexaethylcyclotrisiloxane under the Catalysis of Linear Chlorinated Phosphazene Acid

**DOI:** 10.3390/polym16192835

**Published:** 2024-10-07

**Authors:** Chen Jin, Hao Yang, Yang Zhang, Shuting Zhang, Xu Long, Hong Dong, Yanjiang Song, Zhirong Qu, Chuan Wu

**Affiliations:** College of Material, Chemistry and Chemical Engineering, Key Laboratory of Organosilicon Chemistry and Material Technology, Ministry of Education, Hangzhou Normal University, Hangzhou 311121, China; jinchen@stu.hznu.edu.cn (C.J.); yanghao4@stu.hznu.edu.cn (H.Y.); zhangyang@stu.hznu.edu.cn (Y.Z.); zhangshuting1@stu.hznu.edu.cn (S.Z.); longxu@stu.hznu.edu.cn (X.L.); dh@hznu.edu.cn (H.D.); jsjj_syj@hznu.edu.cn (Y.S.); quzr@hznu.edu.cn (Z.Q.)

**Keywords:** linear chlorinated phosphazene acid, hexaethylcyclotrisiloxane, equilibrium polymerization, silicone fluid, silicone gel

## Abstract

Linear chlorinated phosphazene acid is prepared using PCl_5_ and NH_4_Cl as raw materials. Using hexaethylcyclotrisiloxane as the monomer, 1,1,3,3-tetramethyldisiloxane or 1,3-divinyl-1,1,3,3-tetramethyldisiloxane as the end-capping agent, and linear chlorinated phosphazene acid as the catalyst, polydiethylsiloxane oligomers terminated with active Si-H or Si-CH=CH_2_ groups have been prepared. Using hexaethylcyclotrisiloxane and 1,3,5,7-octamethylcyclotetrasiloxane as comonomers, 1,1,3,3-tetramethyldisiloxane or hexamethyldisiloxane as the end-capping agent, or using hexaethylcyclotrisiloxane and octamethylcyclotetrasiloxane as comonomers, 1,1,3,3-tetramethyldisiloxane as the end-capping agent, copolymers containing active Si-H bonds and dimethylsiloxane segments have been prepared under the catalysis of linear chlorinated phosphazene acid. The effects of catalyst dosage, reaction temperature, reaction time, end-capping agent, and polymerization monomer dosage on polymer yield and structure were investigated. Using 300 ppm of linear chlorinated phosphazene acid, oligomers and copolymers containing an active Si-H bond and diethylsiloxane segment were prepared under mild conditions. The molecular weight of the obtained polymers was close to their designed values, but their PDI values were small. The highest yield of *α*, *ω*-bisdimethylsiloxyl-terminated PDES oligomers reached 93%. Using oligomers and copolymers containing Si-H bonds and diethylsiloxane segments as crosslinkers, a silicone gel containing diethylsiloxane segments was prepared by hydrosilylation reaction. With the introduction of a diethylsiloxane segment, the glass transition temperature of the silicone gel decreased relative to that of the PDMS oligomer, but the temperature at 5% weight loss in nitrogen atmosphere decreased from 347 °C to 312 °C. The mechanism of the ring-opening polymerization of hexaethylcyclotrisiloxane catalyzed by linear chlorinated phosphazene acid is also discussed.

## 1. Introduction

Polysiloxanes with Si-O-Si as the main chain have excellent physical and chemical properties, such as high and low-temperature resistance and thermal stability, due to their high bond energy and large bond angle [1,2,3,4]. The most common polysiloxane is polydimethylsiloxane (PDMS). The two methyl groups attached to Si atoms in PDMS molecules reduce the intermolecular forces, giving it a low surface tension [5,6]. When one or two methyl groups in the main chain of -O-Si(CH_3_)_2_-O- are replaced by other functional substituents, new features are added while the original advantages are retained. After the phenyl group replaces the methyl group, the heat resistance and radiation resistance of the modified silicone materials are excellent, and their refractive index is also significantly improved [7,8,9,10,11,12]. Due to the large electronegativity and electron-withdrawing effect of the fluorine atom, the trifluoropropyl group replacing the methyl group will improve the polymer’s oil resistance, solvent resistance, and lubricity and have a lower surface energy [13,14]. Although the glass transition temperature (*T*g) of PDMS is relatively low [15,16,17], when an ethyl group is introduced, the values of the *T*g of the copolymers will further reduce to −143 °C [18], which makes ethyl-containing polysiloxane materials that have been used in the low-temperature elastomers required for aviation, aerospace, and spacecraft applications for which other synthetic materials are not suitable [18,19,20,21].

In the industry, polydiethylsiloxane (PDES) is mainly prepared using ring-opening polymerization of hexaethylcyclotrisiloxane (D_3_^Et^). However, due to the electron-donating effect of the ethyl group, accomplishing the ring-opening polymerization of the D_3_^Et^ monomer is challenging. It usually requires high temperatures and long reaction times to obtain a higher yield [22,23,24,25,26]. The literature reports that the ring-opening of a D_3_^Et^ monomer can be catalyzed by alkaline catalysts such as KOH [27,28], NaOH [29], alkyl lithium [30,31,32], potassium trimethylsilanolate [33], tetramethylammonium silicate (TMAS) [19,34], or phosphazene superbase [35], or by acidic catalysts such as trifluoromethanesulfonic acid (TfOH) [36] and silicate esters to prepare silicone oil or high molecular PDES oligomers containing diethylsiloxy chain segments and terminated by dimethylvinylsiloxy groups. Among them, researchers [35] have used an organic cyclotriphosphazene base (CTPB) as a catalyst, which can efficiently catalyze the ring-opening polymerization of a D_3_^Et^ monomer and the ring-opening copolymerization of D_3_^Et^ and 1,3,5-trimethyl-1,3,5-tris(3,3,3-trifluoropropyl)cyclotrisiloxane (D_3_^F^) under mild conditions to produce polymers with good low-temperature flexibility and thermal stability.

Like PDMS materials, PDES oligomers or copolymers containing Si-H bonds can be cross-linked with polymers containing Si-CH=CH_2_ functional groups via a platinum-catalyzed hydrosilylation reaction to obtain organosilicon polymers or composite materials with small dimensional changes before and after curing [31]. More importantly, since the hydrosilylation reaction can be completed in the range of 60~150 °C, organosilicon polymers or composite materials containing ethyl functional groups with low-temperature application characteristics can be prepared in situ under mild conditions to meet the needs of multiple industries, such as aviation, aerospace, and electronics [37]. Unfortunately, since Si-H bonds are very prone to dehydrogenation coupling reactions in alkaline environments, only acidic catalysts can be used to prepare such polymers. Even if trifluoromethanesulfonic acid is the strongest acid currently used as a catalyst, the prepared PDES oligomers or copolymers containing Si-H bonds have the defects of small molecular weights and low yields [36].

Although phosphazene superbases have been widely used in the synthesis of various polymers, there is no report in the literature on the synthesis of polymers containing Si-H and diethylsiloxane segments by using phosphazene acid as the catalyst. Since Si-H is incompatible with phosphazene superbases but does not react with acidic phosphazenes, the preparation of acidic phosphazenes and their application in the synthesis of PDES oligomers or copolymers containing Si-H bonds has aroused great interest. To this end, a linear oligomeric chlorophosphazene compound was prepared and used as a catalyst for the ring-opening equilibrium polymerization reaction of cyclosiloxanes to prepare various functionalized polysiloxane fluids. The research disclosed in this manuscript might provide meaningful guidance for the development of hydrogen-containing PDES oligomers or copolymers.

## 2. Experiment Section

### 2.1. Raw Materials

NH_4_Cl, 99.8%, was purchased from Shanghai McLean Biochemical Technology Co., Ltd. (Shanghai, China). 1,2,4-Trichlorobenzene, 99%, was purchased from Sarn Chemical Technology (Shanghai, China) Co., Ltd. (Shanghai, China). Optically pure petroleum ether (boiling range between 60 °C and 90 °C), PCl_5_, dichloromethane (DCM), ethyl acetate (EA), and triethylamine (TEA), all AR grade (purity greater than 99.5%), were purchased from Sinopharm Group Chemical Reagent Co., Ltd. (Shanghai, China). Octamethylcyclotetrasiloxane (D_4_) was purchased from Zhejiang Xin’an Chemical Group Co., Ltd. (Hangzhou, China). 1,1,3,3-Tetramethyldisiloxane (M^H^M^H^), 1,3,5,7-tetramethylcyclotetrasiloxane (D_4_^H^), *α*, *ω*-bisdimethylvinylsiloxyl-terminated PDMS (UC-273-50) with a dynamic viscosity of 50 mPa.s at 25 °C and a vinyl group content of 1.445 wt%, *α*, *ω*-bisdimethylsiloxyl-terminated poly(methylhydrogen-dimethyl)siloxane copolymer (UC-613-47) with a hydrogen content of 0.47 wt%, and *α*, *ω*-bistrimethylsiloxyl-terminated poly(methylhydrogen-dimethyl)siloxane copolymer (UC-203-29) with a hydrogen content of 0.29 wt% were purchased from Jiaxing United Chemical Co., Ltd. (Jiaxing, China). 1,3-divinyl-1,1,3,3-tetramethyldisiloxane (M^Vi^M^Vi^), hexamethyldisilazane (HMDS), was purchased from Jiuding Chemical (Shanghai) Technology Co., Ltd. (Shanghai, China). Hexaethylcyclotrisiloxane (D_3_^Et^) was purchased from Xinyuan Chemical (Shandong, China) Co., Ltd. (Weihai, China). Karstedt’s catalyst, the platinum complex of tetramethyltetravinylcyclotetrasiloxane with a Pt content of 20%, was provided by Heraeus Precious Metals Technology Co., Ltd. (Nanjing, China) and diluted to 0.1% using toluene prior to the hydrosilylation reaction. 1-Ethynyl-1-cyclohexanol (ECH) was purchased from ScienMax Inc. (Plano, TX, USA). All reagents were used directly without further purification.

### 2.2. Experimental Instruments

The dynamic viscosity of each liquid sample at 25 °C was measured using a Brookfield rotational viscometer (DV2TRVTJ0, Brookfield, WI, USA) under a shear rate of 30 s^−1^. The ^1^H NMR spectrum of each oligomer or copolymer was measured using an AVANCEAV 400 nuclear magnetic resonance spectrometer (Bruker, Fällanden, Switzerland). During the test, approximately 10 mg of the liquid sample was dissolved in approximately 0.6 mL of deuterated chloroform (CDCl_3_) with a trace amount of TMS, and the relaxation time D_1_ was set to 5 s. The ^13^C NMR spectrum of each polymer was measured using an AVANCEAV III 500 solid-liquid dual-purpose nuclear magnetic resonance spectrometer (Bruker, Fällanden, Switzerland). During the test, approximately 100 mg of the sample was dissolved in approximately 0.6 mL of CDCl_3_ with a trace amount of TMS, and the relaxation time D_1_ was set to 3 s. The ^29^Si NMR spectrum of each polymer was measured using an AVANCEAV III 500 solid-liquid dual-purpose nuclear magnetic resonance spectrometer (Bruker, Fällanden, Switzerland). During the test, approximately 150 mg of the sample was dissolved in approximately 0.5 mL of CDCl_3_ with a trace amount of TMS, and the relaxation time D_1_ was set to 20 s.

A PL-GPC50 gel permeation chromatograph (Agilent, Santa Clara, CA, USA) equipped with a StyragleHT2 toluene separation column was used to test the weight average molecular weight (*M*w), number average molecular weight (*M*n), and polydispersity index (PDI) of each sample. The test column temperature was set to 25 °C. Toluene, with a flow rate of 1.0 mL/min, was used as the mobile phase, and PDMS with a known molecular weight (490, 6200, 15,000, 34,100, 64,800, 95,800, 219,400, 384,500, and 610,000 g/mol, American Polymer Standards Corporation, OH, USA) was used as the reference material.

A Discovery TGA thermogravimetric analyzer (TA Instruments Ltd., New Castle, DE, USA) was used to test the sample’s thermal stability in a nitrogen atmosphere. The test temperature range was between 40 and 800 °C, the carrier gas flow rate was 15 mL/min, and the heating rate was 20 °C/min. The samples’ FT-IR spectra were recorded on a Bruker Alpha-T (Ettlingen, Germany) in the range of 4000 to 400 cm^−1^ at a resolution of 4 cm^−1^ and scanned 64 times. DSC curves were recorded on a Netzsch DSC 214 (Netzsch, Wittelsbacherstrasse, Germany) instrument in a nitrogen atmosphere at a flow rate of 40 mL/min. During the test, the temperature was raised from 40 °C to 200 °C at a heating rate of 10 °C/min, then cooled from 200 °C to −150 °C at a rate of −10 °C/min, and then heated from −150 °C to 200 °C at a heating rate of 10 °C/min to obtain the DSC curves of the cooling stage and the heating stage.

### 2.3. Preparation of Linear Chlorinated Phosphazene Acid

**Scheme 1:**
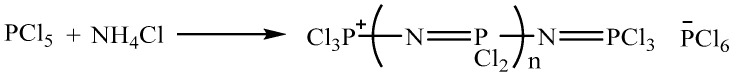
Preparation of linear chlorinated phosphazene acid catalyst.

According to the scheme shown in Scheme 1, 41.65 g (0.2 mol) of PCl_5_, 5.35 g (0.1 mol) of NH_4_Cl, 124.6 mL of 1,2,4-trichlorobenzene, and 0.21 g of anhydrous MgCl_2_ were added into the reactor and refluxed for 8 h. The reaction solution was then poured into petroleum ether to precipitate a pale yellow solid. The solid was recovered by vacuum filtration to obtain 32.2 g of a light yellow solid, with a yield of 69%. When the catalyst was used in subsequent experiments, it was diluted to a mass fraction of 2.5 wt% with DCM to facilitate weighting.

### 2.4. Synthesis of Linear PDES Oligomers and Copolymers

Linear chlorinated phosphazene acid was used as the catalyst to investigate its influence on the equilibrium polymerization of cyclosiloxanes, especially D_3_^Et^, to prepare Si-H or Si-CH=CH_2_ functionalized PDES oligomers and copolymers.

**Scheme 2:**
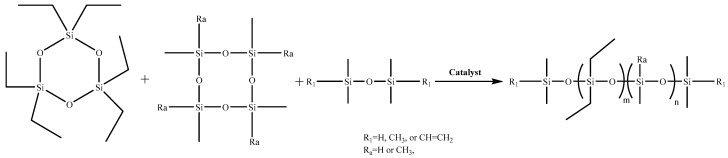
Synthesis of linear PDES oligomer or copolymer terminated with different groups.

When using M^Vi^M^Vi^ as the end-capping agent, D_3_^Et^ as the monomer, and HDMS as the passivator, an *α*, *ω*-bisdimethylvinylsiloxyl-terminated PDES oligomer (PDES-Vi) could be synthesized according to Scheme 2. In a 100 mL three-necked flask, 39.38 g (0.1284 mol) of D_3_^Et^ and 7.98 g (0.0428 mol) of M^Vi^M^Vi^ were added in sequence, and stirred thoroughly to mix evenly. Then 500 ppm of linear chlorinated phosphazene acid catalyst was added. The mixture was then reacted at 90 °C for 8 h. After the reaction solution was cooled to room temperature, the passivator HDMS was added and stirred evenly to deactivate the catalyst. After removing the solid by filtration, the filtrate was distilled under reduced pressure to remove low-boiling components, and 35.87 g of a clear and transparent vinyl-terminated linear PDES oligomer (PDES-Vi) was obtained with a yield of 76%.

When using MM or M^H^M^H^ as the end-capping agent and D_3_^Et^ and/or D_4_ or D_4_^H^ as the monomer, *α*, *ω*-bisdimethylsiloxyl-terminated PDES oligomers (PDES-H), α, ω-bisdimethylsiloxyl-terminated poly(methylhydrogen-diethyl)siloxane copolymers (PMHS-*co*-PDES), *α*, *ω*-bistrimethylsiloxyl-terminated PMHS-*co*-PDES copolymers, and *α*, *ω-*bisdimethylsiloxyl-terminated poly(dimethyl-diethyl)siloxane copolymers (PDMS-*co*-PDES) could be obtained using a procedure similar to that used to produce a PDES-Vi oligomer.

In the process of synthesizing these polymers, certain amounts of D_3_^Et^ and/or D_4_ or D_4_^H^ and the end-capping agent (MM or M^H^M^H^) were added to a three-necked flask and stirred thoroughly to mix all ingredients evenly. Then, a linear chlorine phosphazene acid catalyst was added, and the reaction was conducted at a temperature for a certain period. The reaction solution was then cooled to room temperature. After the passivating agent was added, the mixture was stirred evenly to deactivate the catalyst. The filtrate was collected by filtration to remove the solid and distilled under reduced pressure to remove low-boiling materials, obtaining a clear and transparent functionalized linear PDES oligomer or copolymer. Detailed information on synthesizing these polymers is presented in Table 1, Table 2, Table 3 and Table 4.

### 2.5. Preparation of Silicone Gels

The prepared *α*, *ω*-bistrimethylsiloxyl- or *α*, *ω*-bisdimethylsiloxyl-terminated PMHS-*co*-PDES containing active Si-H bonds or commercially available *α*, *ω*-bistrimethylsiloxyl- or *α*, *ω*-bisdimethylsiloxyl-terminated PDMS functioned as the cross-linking agent, blended with PDES-Vi fluid in a molar ratio of Si-H/Si-CH=CH_2_=1.4:1. Meanwhile, 10 ppm of Karstedt’s catalyst and 10 ppm of ECH used as an inhibitor were added, stirred evenly to remove bubbles under vacuum. When these mixtures were placed in a thermostat oven at 80 °C for 1 h, the cross-linked silicone gel samples containing Et_2_SiO segments were prepared.

When the *α*, *ω*-bistrimethylsiloxyl- or *α*, *ω*-bisdimethylsiloxyl-terminated PMHS-*co*-PDES were replaced by *α*, *ω*-bistrimethylsiloxyl-terminated PMHS-*co*-PDMS and an *α*, *ω*-bisdimethylvinylsiloxyl-terminated PDMS oligomer is used to replace the *α*, *ω*-bisdimethylvinylsiloxyl-terminated PDES oligomer, a silicone gel containing only Me_2_SiO segments was produced according to the method described above. This gel was used as the control sample.

## 3. Results and Discussion

Significantly affected by ring tension, cyclotrisiloxane with the same functional group attached to Si atoms is more active than cyclotetrasiloxane [35]. Compared with D_4_, of which the functional groups attached to Si atoms are methyl groups, the ring-opening polymerization activity of D_3_^Et^ is far inferior to that of D_4_ due to the influence of the electron-donating effect of the ethyl group, which results in a lower yield of PDES oligomers or copolymers containing Et_2_SiO segments. In the literature [28], KOH is usually used as a catalyst, and a reaction temperature as high as 140 °C or even higher is used to partially achieve a ring-opening polymerization of D_3_^Et^ to obtain a silicone polymer containing Et_2_SiO segments. However, silicone polymers containing Et_2_SiO segments whose functional groups contain Si-H groups can undergo a severe dehydrogenation coupling reaction and release a large amount of H_2_, because Si-H bonds react violently with alkaline substances. Therefore, under the current catalytic conditions, a polymer containing both Si-H bonds and Et_2_SiO segments has never been reported before.

### 3.1. PDES Oligomers

#### 3.1.1. α, ω-bisdimethylsiloxyl-Terminated PDES Oligomers (PDES-H)

In order to investigate the effect of a linear chlorinated phosphazene acid catalyst on the ring-opening polymerization of D_3_^Et^, D_3_^Et^ was used as the monomer, M^H^M^H^ was used as the end-capping agent, and HDMS was used as the passivator to prepare a PDES-H fluid. The effects of reaction temperature, reaction time, and catalyst dosage on the structure and yield of the polymerization product were explored. The results are shown in Table 1.

Taking Entry 1C in Table 1 as an example, the ^1^H NMR spectrum, ^13^C NMR spectrum, and ^29^Si NMR spectrum of the prepared PDES-H are shown in Figure S1a–c in the Supplementary Materials.

One could find from Figure S1a in the Supplementary Materials that the peak at about *δ* = 0.2 ppm is the proton signal of all of the methyl groups (C***H***_3_-Si) bonded to the Si atoms, and the signal at about *δ =* 4.7 ppm is attributed to the proton in the Si-***H*** bond. At about *δ* = 0.5 ppm, the proton signal in the repeating unit of Si-C***H***_2_CH_3_ could be clearly observed. The proton signal of Si-CH_2_C***H***_3_ is observed at about *δ* = 0.9 ppm. The chemical shift at about *δ* = 0.0 ppm is the trace amount of TMS contained in CDCl_3_. In addition to these signals, no other impurity peak signals were observed, as shown in Figure S1a. The ^13^C NMR spectrum of Entry 1C is shown in Figure S1b of the Supplementary Materials. The chemical shift at *δ =* 0.7 ppm is attributed to the characteristic peak of Si-***C***H_3_, the chemical shift at *δ* = 6.5 ppm is the characteristic peak of the repeating unit Si-***C***H_2_CH_3_, and the chemical shift at *δ =* 7.3 ppm is the characteristic peak of the repeating unit Si-CH_2_***C***H_3_. No other impurity peak signals could be observed, as shown in Figure S1b. The ^29^Si NMR spectrum of Entry 1C is shown in Figure S1c of the Supplementary Materials. The chemical shift at *δ =* −7.4 ppm is attributed to the characteristic peak of -O-***Si***(CH_3_)_2_H. The chemical shift at *δ* = −22.9 ppm is the characteristic peak of the repeating unit -O-***Si***(CH_2_CH_3_) _2_-O-. The chemical shift at *δ =* −20.78 ppm is attributed to the silicon signals in the Et_2_SiO segments of PDES-H oligomers with a lower degree of polymerization. The results shown in Figure S1a–c indicate that the target polymer was successfully synthesized.

The method used to calculate the degree of polymerization (*m*_exp_) and the molecular weight of the polymer (*M*_NMR_) from the ^1^H NMR spectrum is listed in Section S2.1 of the Supplementary Materials, and the results are presented in Table 1.

**Figure 1:**
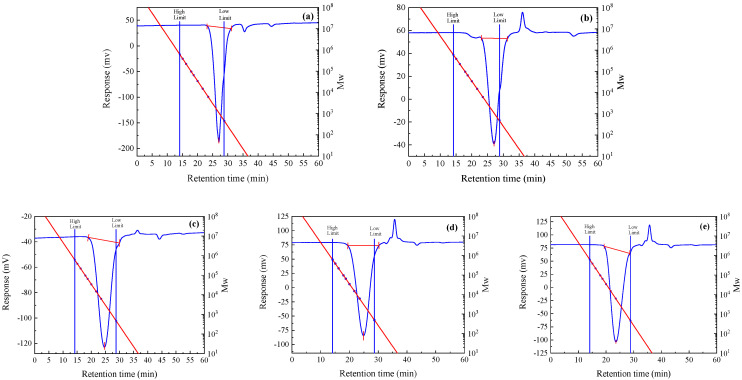
GPC curve of PDES oligomers and copolymers ((**a**) PDES-H, Entry 1C in Table 1; (**b**) PDES-Vi; (**c**) *α*, *ω*-bisdimethylsiloxyl-terminated PMHS-*co*-PDES, Entry 2A in Table 2; (**d**) *α*, *ω-*bistrimethylsiloxyl-terminated PMHS-*co*-PDES, Entry 3A in Table 3; (**e**) *α*, *ω-*bisdimethylsiloxyl-terminated PDMS-*co*-PDES, Entry 4A in Table 4).

Figure 1a shows the GPC curve of a PDES-H sample (Table 1, Entry 1C). The polydispersity index PDI = 1.46 was determined from the GPC curve. The GPC curve shows the typical normal distribution characteristics of polymers, with only one prominent peak present, thus confirming that the sample is a single-component polymer. The GPC test results of other PDES samples are provided in Table 1.

As can be seen from Table 1, when the catalyst concentration is 500 ppm and the reaction temperature is 50 °C, the yield of the obtained polymer is only 50% after 5 h of reaction, and the PDI value of the polymer is 1.46. When the reaction time is extended to 7 h, the yield of the polymer reaches 93% and its PDI value is 1.50. When the reaction time is further extended to 9 h, the yield of the polymer remains unchanged and the PDI value fluctuates slightly. When the reaction time is further extended to 11 h, the yield of the polymer decreases slightly. It can be seen that when preparing PDES oligomers end-capped with dimethylsiloxyl group at a reaction temperature of 50 °C, the suitable reaction time is 7 to 9 h.

The reaction used to prepare oligomers via the ring-opening polymerization of D_3_^Et^ catalyzed by phosphazene acid is a fascinating equilibrium polymerization reaction. When PDMS materials are prepared by equilibrium polymerization under acidic catalyst conditions using raw materials containing dimethylsiloxy segments, such as D_4_ or a mixture of dimethylcyclosiloxanes (DMC), as monomers [25], there is a delicate interplay between monomer ring-opening polymerization and polymer depolymerization. This results in the yield of the equilibrium polymerization reaction being maintained at around 90%, a phenomenon consistent with the experimental observations reported in this manuscript. It can also be seen from Table 1 that when the catalyst dosage is 500 ppm and the reaction time is 9 h, relative to the product obtained at other temperatures, the molecular weight distribution of the product obtained at 50 °C (Table 1, Entry 1C) is the lowest (1.46). Its appearance is a clear and transparent liquid, and the yield is also the highest. The reason for this might be related to the low boiling point (70 °C) of M^H^M^H^. The higher the reaction temperature, the more volatile the M^H^M^H^ will be, resulting in a decrease in the amount of the end-capping agent and an increase in the viscosity of the synthesized PDES oligomers (Table 1, Entry 1H). When the temperature continues to increase, the highly acidic linear chlorinated phosphazene quickly catalyzes the degradation of the PDES oligomer, causing the polymer viscosity to decrease, accompanied by similar changes in the *M*n (Table 1, Entry 1I, and Entry 1J). The PDI distribution of these polymers widens with the increase in temperature, which might be related to the compositional changes in the reaction mixture caused by the volatilization of M^H^M^H^.

The effect of catalyst concentration on the reaction results can also be observed in Table 1. For example, when the reaction temperature is 60 °C and the reaction time is 9 h (Table 1, Entries 1E–1H), as the catalyst dosage decreases from 500 ppm (Table 1, Entry 1H) to 300 ppm (Table 1, Entry 1F), the PDI values of the PDES decreased from 1.69 to 1.42. The yield increased to 88% at a catalyst concentration of 300 ppm, indicating that reducing the catalyst dosage can alleviate the degradation side reactions of PDES.

When the catalyst concentration was further reduced to 200 ppm (Table 1, Entry 1E), after 9 h of reaction at 60 °C, the yield of PDES-H fluid was reduced to 50%. Meanwhile, its PDI value increased to 1.66, indicating that the amount of catalyst was insufficient at this time. On the one hand, not all of the D_3_^Et^ could be converted to PDES oligomers of different molecular weights via ring-opening polymerization. On the other hand, at this catalyst concentration, the PDES oligomers of different molecular weights present in the system cannot be rearranged to obtain PDES-H oligomers with a high yield and well-ordered segment structure.

Therefore, when using linear chlorinated phosphazene acid as a catalyst to prepare PDES-H oligomers, a liquid with the highest yield, narrow molecular weight distribution, and clear and transparent appearance can be achieved when the reaction is conducted at a temperature of 50 to 60 °C for 7−9 h under a catalyst concentration of 300 ppm.

#### 3.1.2. *α*, *ω*-bisdimethylvinylsiloxyl-Terminated PDES Oligomers (PDES-Vi)

The kinetic viscosity of the PDES-Vi oligomer was measured using a rotational viscometer, and the result was 73.75 mPa.s. The structure of the product was analyzed using ^1^H NMR, ^13^C NMR, ^29^Si NMR, and FT-IR, and the results are presented in Figure S2 of the Supplementary Materials. The degree of polymerization (*m*), molecular weight (*M*_NMR_), and vinyl content of the oligomer were calculated from the area integration value of each proton in the ^1^H NMR spectrum. The results were *m* = 13, *M*_NMR_ = 1512 g/mol, and the content of vinyl groups was 3.57wt%, as illustratively shown in Section S2.2 of the Supplementary Materials.

As can be seen from Figure S2a, the peak at about *δ =* 0.15 ppm is the proton signal on all of the methyl groups (Si-C***H***_3_) bonded to Si atoms. The multiple peaks at about *δ* = 6.0 ppm are attributed to the proton signal of the Si-C***H***=C***H***_2_ group. The chemical shift at *δ* = 0.5 ppm is the proton signal of the repeating unit Si-C***H***_2_CH_3_ and the chemical shift at *δ =* 0.9 ppm is the proton peak of the repeating unit Si-CH_2_C***H***_3_. No other apparent impurity peaks can be observed in the ^1^H NMR spectrum. The ^13^C NMR spectrum of PDES-Vi is shown in Figure S2b of the Supplementary Materials. The chemical shift at *δ* = 0.0 ppm is the characteristic peak of Si-***C***H_3._ The chemical shift at *δ =* 6.3 ppm is the characteristic peak of the repeating unit Si-***C***H_2_CH_3_. The chemical shift at *δ =* 7.2 ppm is the characteristic peak of the repeat unit Si-CH_2_***C***H_3_. The chemical shifts at *δ =* 131.2 ppm and *δ =* 139.2 ppm are the characteristic peaks of C atoms in the terminal Si-***C***H=***C***H_2_ group. Meanwhile, no other apparent impurity peaks could be found in the spectrum. As can be seen from Figure S2c, the chemical shift at *δ =* −4.9 ppm is the characteristic peak of -O-***Si***(CH_3_)_2_CH=CH_2._ The chemical shift at *δ =* −22.9 ppm is the characteristic peak of the repeating unit -O-***Si***(CH_2_CH_3_) _2_-O-.

As can be seen from Figure S2d, there is a C-H stretching vibration absorption peak at 2956 cm^−1^, attributed to Si-CH_3_, and a weak C-H stretching vibration absorption peak at 3052 cm^−1^, attributed to Si-CH=CH_2_ [38]. The signal that appears at 1592 cm^−1^ belongs to the C=C stretch of the terminal methylene group of the vinyl group [38,39,40,41,42], and the signal that appears at 1408 cm^−1^ is part of the =CH_2_ scissors within the vinyl group [41,42]. Since this signal is very intense and the bands located between 1400 and 1500 cm^−1^ are primarily associated with vibrations within the saturated alkyl groups, the overlap of the =CH_2_ scissors within the vinyl group and the vibrations within the saturated alkyl groups provide major contributions to this signal. The peaks at 1243 cm^−1^ and 784 cm^−1^ are the Si-CH_3_ stretching vibration absorption peaks in siloxane, and the peak at 998 cm^−1^ is the stretching vibration of the Si-O-Si vibration peak. The characteristic peak of Si-CH_2_CH_3_, which should appear at 1235 cm^−1^, was not observed, which might be the result of the influence of Si-CH_3_.

Figure 1b shows the GPC curve of the prepared sample. The values of *M*n and PDI, determined by GPC using PDMS with known molecular weights as a reference, are 800 g/mol and 1.69, respectively. Except for a tiny peak observed near the retention time of 21 min, the GPC curve of PDES-Vi only showed one peak near the retention time of 27 min. The former is a polymer impurity with a high degree of polymerization generated by a longer reaction time.

### 3.2. Poly(methylhydrogen-diethyl)siloxane Copolymers (PMHS-co-PDES)

Since PDES has the disadvantages of forming rigid crystals at low temperatures and disordered crystal conformation at high temperatures [16], the application prospects of PDES in the field of low-temperature elasticity are limited. Once other structure units, such as the MeHSiO segment, Me_2_SiO segment, MePhSiO segment, or Me(CH_2_CH_2_CF_3_)SiO segment [43], are introduced into the chain segments of PDES, the low-temperature crystallization behavior of PDES can be significantly inhibited. However, the ring-opening polymerization reaction rates of D_3_^Et^ and other cyclosiloxanes are pretty different, and it is not easy to obtain copolymers with uniform structures using traditional catalytic systems [22,44]. After investigating the ring-opening polymerization of D_3_^Et^ catalyzed by linear chlorinated phosphazene acid, the effect of this catalyst on the ring-opening copolymerization process of mixed cyclosiloxanes was further explored.

#### 3.2.1. *α*, *ω*-bisdimethylsiloxyl-Terminated PMHS-co-PDES

According to the synthesis route shown in Scheme 2, D_3_^Et^ and D_4_^H^ are used as the monomers, and HDMS is used as the passivator, using M^H^M^H^ as the end-capping agent to prepare an *α*, *ω-*bisdimethylsiloxyl-terminated PMHS-*co*-PDES fluid. The raw materials used and the results obtained are shown in Table 2.

Figure S3a–c shows the ^1^H NMR, ^13^C NMR, and ^29^Si NMR spectra of the *α*, *ω*-bisdimethylsiloxyl-terminated PMHS-*co*-PDES (Table 2, Entry 2A). The copolymer has repeated MeHSiO and Et_2_SiO segments, and the Si-H bond is both located at the terminal group and attached to Si atoms. 1, 4-dioxane can be used as an external standard to calculate the degree of polymerization of each repeating unit. As can be seen in Figure S3a, the peak at about *δ =* 0.16 ppm is the proton signal of all methyl groups (C***H***_3_-Si) bonded to the Si atom, and the peak at about *δ =* 4.7 ppm is the proton signal of Si-***H***. The chemical shift at about *δ =* 0.5 ppm is the proton peak of the repeating unit Si-C***H***_2_CH_3_, and the chemical shift at about *δ =* 0.9 ppm is the proton peak of the repeating unit Si-CH_2_C***H***_3_. The chemical shift at *δ =* 3.7 ppm is attributed to the protons of 1, 4-dioxane. Apart from the proton signals mentioned above and a single peak at *δ =* 0.0 ppm, which results from a trace amount of TMS in CDCl_3_, no other prominent peaks could be observed. The method of calculating the degree of polymerization of the Et_2_SiO segments (*m*), the degree of polymerization of the MeHSiO segments (*n*), and the molecular weight of the polymer (*M*_NMR_) from the ^1^H NMR spectrum is listed in Section S2.3 of the Supplementary Materials, and the results are presented in Table 2.

As can be seen from Figure S3b, the carbon atom signal near *δ =* 0.7 ppm is the characteristic peak of Si-***C***H_3_. The carbon atom signal near *δ =* 6.4 ppm is attributed to the characteristic peak of Si-***C***H_2_CH_3_ in the repeating unit. The carbon atom signal near *δ =* 7.3 ppm is the characteristic peak of Si-CH_2_***C***H_3_ in the repeating unit. In the ^13^C NMR spectrum, except for the above carbon atom signals, there are no other prominent peaks. As can be seen from Figure S3c, the silicon atom signal near *δ =* −6.7 ppm is the characteristic peak of -O-***Si***(CH_3_)_2_H. The silicon atom signal near *δ =* −21.0 ppm is attributed to the characteristic peak of -O-***Si***(CH_2_CH_3_) _2_-O- in the repeating unit. The silicon atom signal near *δ =* −36.9 ppm is the characteristic peak of -O-***Si***(H)-CH_3_-O- in the repeating unit. In the ^29^Si NMR spectrum, except for the above silicon atom signals, there are no other prominent peaks. These spectra indicate that the target copolymer has been successfully synthesized. The ^1^H NMR spectra and ^13^C NMR spectra of other *α*, *ω-*bisdimethylsiloxyl-terminated PMHS-*co*-PDES samples are shown in Figures S4–S7.

Taking Entry 2A in Table 2 as an example, its typical GPC curve is shown in Figure 1c. The values of the average molecular weight of this copolymer, *M*n = 3000 g/mol, and polydispersity index PDI=1.98 were determined from the GPC curve. The GPC curve of the sample shows the typical normal distribution characteristics of polymers, and there is only one prominent peak to be observed, which is consistent with the characteristics of a single-component polymer. The GPC curves of other *α*, *ω*-bisdimethylsiloxyl-terminated PMHS-*co*-PDES samples are shown in Figures S4–S7. The values of *M*n and PDI determined from the GPC curves are listed in columns 12 and 13 of Table 2, respectively.

#### 3.2.2. *α*, *ω*-bistrimethylsiloxyl-Terminated PMHS-co-PDES

According to the synthesis route shown in Scheme 2, D_3_^Et^ and D_4_^H^ are used as the copolymerization monomers. MM was used as the end-capping agent, and linear chlorinated phosphazene acid was used as the catalyst. HDMS was used as the passivator to prepare *α*, *ω-*bistrimethylsiloxyl-terminated PMHS-*co*-PDES. The raw materials used and the results obtained are shown in Table 3.

Taking Entry 3A in Table 3 as an example, its ^1^H NMR spectrum, ^13^C NMR spectrum, and ^29^Si NMR spectrum are shown in Figure S8a–c. As can be seen in Figure S8a, the peak at about *δ* = 0.0 ppm is attributed to the trace amount of TMS in CDCl_3_ solvent. The proton signal at about *δ* = 0.1 ppm results from the protons on all methyl groups (C***H***_3_-Si) bonded to Si atoms. The peak at about *δ* = 4.7 ppm is the proton signal of Si-***H***. The chemical shift at *δ* = 0.5 ppm is attributed to the proton signal of the repeating unit Si-C***H***_2_CH_3_, and the chemical shift at *δ* = 0.9 ppm is the proton signal of the repeating unit Si-CH_2_C***H***_3_. The chemical shift at *δ = *3.7 ppm is attributed to the proton signals of 1, 4-dioxane. Except for the proton signals mentioned above, no other prominent peaks could be observed. From ^1^H NMR and the structure of the copolymer, the values of the polymerization degree *m* of the Et_2_SiO segments and the polymerization degree *n* of the MeHSiO segments in each copolymer can be calculated. After that, the molecular weight *M*_NMR_ of each copolymer can further be calculated. The detailed calculation process is presented in Section S2.4 in the Supplementary Materials.

As can be seen from Figure S8b, the chemical shift at about *δ =* 0.7 ppm is the characteristic peak of Si-***C***H_3_; the chemical shift at *δ =* 6.4 ppm is the characteristic peak of the repeating unit Si-***C***H_2_CH_3_; the chemical shift at *δ =* 7.3 ppm is the characteristic peak of the repeating unit of Si-CH_2_C***H***_3_. Except for the above carbon atom signals, no other apparent signals were observed in the ^13^C NMR spectrum. As can be seen from Figure S8c, the chemical shift at *δ =* 7.1 ppm is the characteristic peak of -O-***Si***(CH_3_)_3_. The chemical shift at *δ =* −20.9 ppm is the characteristic peak of the repeating unit -O-***Si***(CH_2_CH_3_) _2_-O-. The chemical shift at *δ =* −37.5 ppm is the characteristic peak of the repeating unit of -O-***Si***(H)-CH_3_-O-. Except for the silicon atom signals described above, no other apparent signals were observed in the ^29^Si NMR spectrum. The above results indicate that the target polymer has been successfully synthesized. The ^1^H NMR spectra and ^13^C NMR spectra of other *α*, *ω-*bistrimethylsiloxyl-terminated PMHS*-co-*PDES samples are shown in Figures S9–S11.

The GPC curve of the prepared *α*, *ω-*bistrimethylsiloxyl-terminated PMHS-*co*-PDES (Entry 3A in Table 3) is listed in Figure 1d. The average molecular weight *M*n and the polydispersity index (PDI) determined from the GPC curve were *M*n = 2200 g/mol and PDI = 2.30. The average molecular weight *M*n and PDI of other samples determined from their GPC curves (Figures S9c, S10c and S11c in the Supplementary Materials) are listed in the 11th and 12th columns of Table 3, respectively.

### 3.3. Poly(dimethyl-diethyl)siloxane Copolymer (PDMS-co-PDES)

As illustrated in Scheme 2, when D_3_^Et^, and D_4_ are used as the monomers, HDMS is used as the passivator, and M^H^M^H^ is used as the end-capping agent to explore the preparation method of *α*, *ω-*bisdimethylsiloxyl-terminated PDMS-*co*-PDES catalyzed by linear chlorinated phosphazene acid. The results are shown in Table 4.

The ^1^H NMR spectrum, ^13^C NMR spectrum, and ^29^Si NMR spectrum of the prepared *α*, *ω-*bisdimethylsiloxyl-terminated PDMS-*co*-PDES sample (Entry 4A in Table 4) are shown in Figure S12a–c. As can be seen from Figure S12a, the peak at about *δ = *0.1 ppm is the proton peak on all methyl groups (C***H***_3_-Si) bonded to Si atoms. The chemical shift at about *δ = *4.7 ppm is the proton signal of Si-***H***. The chemical shift at *δ = *0.5 ppm is the proton peak of methylene in the repeating unit Si-C***H***_2_CH_3_, and the chemical shift at *δ = *0.9 ppm belongs to the proton signal of methyl group in the repeating unit Si-CH_2_C***H***_3_. In addition to the proton signals mentioned above, no other apparent impurity peak signals were observed in the ^1^H NMR spectrum. From ^1^H NMR and the structure of the copolymer, values of the polymerization degree *m* of the Et_2_SiO segments and the polymerization degree *n* of the Me_2_SiO segments in each copolymer can be calculated. After that, the molecular weight *M*_NMR_ of each copolymer can further be calculated. The detailed calculation process is presented in Section S2.5 of the Supplementary Materials.

As shown in Figure S12b, the chemical shift at about *δ =* 0.8 ppm is the characteristic peak of Si-***C***H_3_. The chemical shift at *δ =* 6.5 ppm is the characteristic carbon atom signal in the repeating unit Si-***C***H_2_CH_3_. The chemical shift at *δ =* 7.3 ppm belongs to the characteristic carbon atom signal in the repeating unit Si-CH_2_***C***H_3_. Except for the carbon atom signal mentioned above, no other apparent impurity peaks were observed in the spectrum. As can be seen from Figure S12c, the chemical shift at *δ =* −7.1 ppm is the characteristic peak of -O-***Si***(CH_3_)_2_H. The chemical shift at *δ =* −21.9 ppm is the characteristic peak of the repeating unit -O-***Si***(CH_2_CH_3_)_2_-O-. The chemical shift at *δ =* −23.2 ppm is the characteristic peak of the repeating unit of -O-***Si***(CH_3_)_2_-O-. Except for the silicon atom signals mentioned above, no other apparent signals were observed in the ^29^Si NMR spectrum. The above spectra indicate that the target polymer was successfully synthesized. The ^1^H NMR spectra and ^13^C NMR spectra of other *α*, *ω-*bisdimethylsiloxyl-terminated PDMS-*co*-PDES samples are shown in Figures S13–S16.

The GPC curve of the prepared sample (Entry 4A in Table 4) is shown in Figure 1e. The measured *M*n is 3900 g/mol, and PDI is 1.88. The sample’s GPC curve shows that the product has a single peak with a high molecular weight and exhibits a standard distribution characteristic. Meanwhile, only one prominent peak exists. These analysis data indicate that the sample is a single-component polymer. The GPC curves of other *α*, *ω-*bisdimethylsiloxyl-terminated PDMS-*co*-PDES samples are shown in Figures S13–S16. The number average molecular weight *M*n value and PDI value of each sample determined from the GPC curve are listed in the 12th and 13th columns of Table 4, respectively.

From Table 2, Table 3 and Table 4 and the analysis results mentioned above, it can be seen that when linear chlorinated phosphazene acid is used as the catalyst and D_3_^Et^, D_4_^H^, or D_4_ are used as the monomers, PDMS-*co*-PDES or PMHS-*co*-PDES containing active Si-H bonds can be successfully prepared. The polymer has a narrow molecular weight distribution and is a clear and transparent liquid. When the catalyst dosage is 200 ppm, the monomers can be copolymerized, but the polymerization yield is low. Increasing the catalyst dosage can increase the polymerization yield. To the best of our knowledge, this is the first report on synthesizing PDES polymers containing active Si-H bonds using an acidic chlorinated phosphazene catalyst and gaining a much higher yield than that of using TfOH as the catalyst [36].

### 3.4. Effect of Ethyl Content on Heat Performance of Silicone Gels

In order to investigate the influence of ethyl content on the thermal properties of a silicone gel, the synthesized PDES-Vi oligomer or commercially available *α*, *ω*-bisdimethylvinylsiloxyl-terminated polydimethylsiloxane oligomer (UC-273-50) is used as the matrix. The synthesized *α*, *ω*-bisdimethylsiloxyl-terminated PMHS-*co*-PDES (Table 2, Entry 2B) or *α*, *ω*-bistrimethylsiloxyl-terminated PMHS-*co*-PDES (Table 3, Entry 3B), or commercially available *α*, *ω*-bisdimethylsiloxyl-terminated (UC-613-47) or *α*, *ω*-bistrimethylsiloxyl-terminated PMHS-*co*-PDMS (UC-203-29) functions as the cross-linking agent. When the molar ratio of Si-H and Si-CH=CH_2_ was adjusted to 1.4:1, a series of silicone gels was prepared according to the method described in Section 2.4. The structures and amounts of the Si-CH=CH_2_ containing siloxane polymers and those of Si-H containing siloxane polymers are presented in Table 5. The ethyl content in each silicone gel is determined by area integration from the ^1^H NMR spectra of the mixture consisting of the Si-H and Si-CH=CH_2_ constituents without the addition of Karstedt’s catalyst. The results of the measurements of the ethyl content and the thermal properties of each vulcanized silicone gel measured by DSC and TGA are also listed in Table 5.

#### 3.4.1. Structural Analysis of Silicone Gels

**Figure 2:**
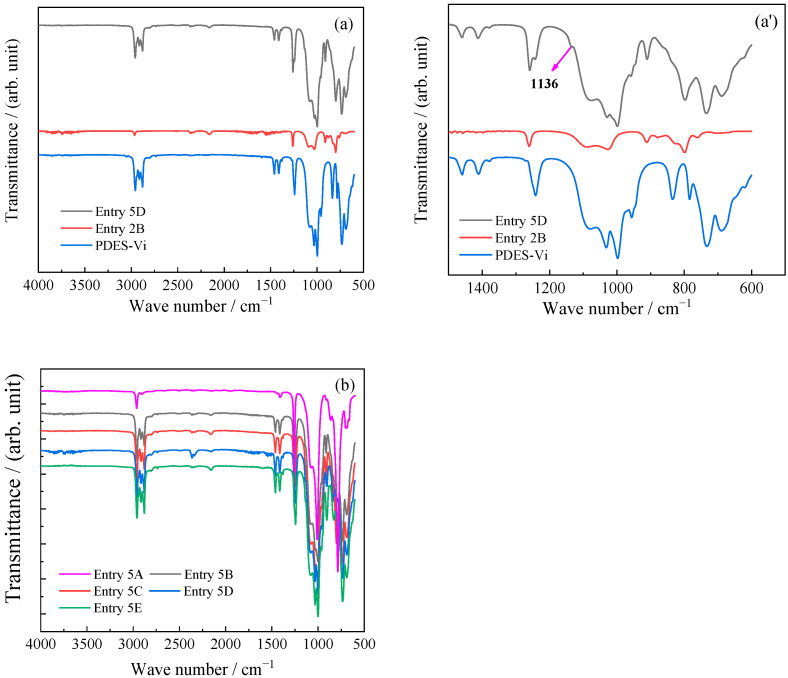
FT-IR spectra of silicone gels. (**a**) comparison of FT-IR spectra before and after curing of silicone gel; (**a’**) partially enlarged view of Figure 2a between 1500 and 500 cm^−1^; (**b**) FT-IR spectra of all silicone gels.

Taking Entry 5D in Table 5 as an example, a comparison of the FT-IR spectra before and after the silicone gel is prepared is shown in Figure 2a, and the FT-IR spectra of all of the silicone gel samples are shown in Figure 2b. As can be seen from Figure 2a, the bending vibration absorption peak attributed to excess Si-H bonds in the silicone gel sample could be observed at wave numbers of 2159 cm^−1^ and 911 cm^−1^, because the molar ratio of Si-H to Si-CH=CH_2_ was designed to be 1.4:1. Meanwhile, a new characteristic absorption peak attributed to C-C stretching vibration was observed at 1136 cm^−1^ [45,46], together with the disappearance of the stretching vibration peak attributed to the C=C double bond at 1597 cm^−1^, which indicates that the hydrosilylation reaction between C=C and Si-H occurred and the silica gel was successfully prepared.

As can be seen from Figure 2b, the C-H stretching vibration peak of these five silicone gel samples was observed at a wave number of 2954 cm^−1^. The C-H bending vibration peak was observed at a wave number of 1416 cm^−1^ and the Si-O-Si stretching vibration peak was observed at a wave number of 1031 cm^−1^, together with the stretching vibration absorption peaks at 1253 cm^−1^ and 783 cm^−1^ attributed to the Si-CH_3_ bond in siloxane. Affected by Si-CH_3_, no characteristic peak attributed to Si-CH_2_CH_3_ was observed at the wave number of 1236 cm^−1^.

#### 3.4.2. Thermal Properties of Silicone Gel Samples

##### DSC Curves

**Figure 3:**
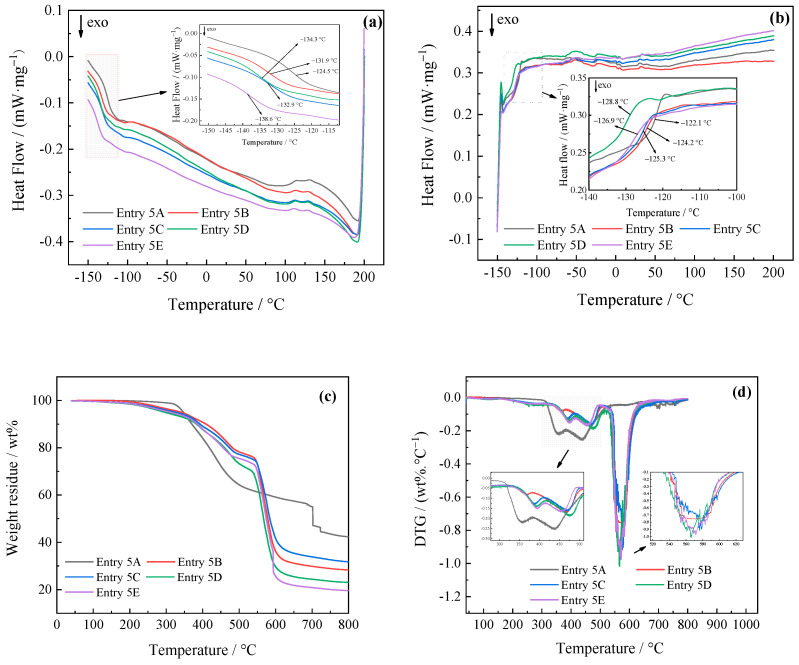
Thermal performances of silicone gel samples. (**a**) DSC curves in temperature decline stage; (**b**) DSC curves in temperature increasing stage; (**c**) TGA curves; (**d**) DTG curves.

Each silicone gel sample was subjected to DSC testing, and the obtained DSC curves of the cooling stage and the heating stage are shown in Figure 3a,b. As Figure 3a shows, no prominent crystallization peak was observed for all of the silicone gel samples investigated within the test range. Figure 3a, b also show that each silicone gel sample has good structural stability in the temperature range of −120 °C to 100 °C, and no apparent crystallization peaks and melting peaks could be observed in this temperature range.

*T*g is an essential parameter for evaluating the performance of polymer materials or composites. It can be obtained either from the cooling [47] or heating curve [33,35] in a DSC analysis. In the cooling stage (Figure 3a), the glass transition temperature of these silicone gel samples can be observed in the precise range of −138.6~−124.5 °C, and in the heating stage (Figure 3b), it is within the specific range of −128.8 °C~−122.1 °C. These specific temperature ranges provide detailed insights into the behavior of the materials and are consistent with the low-temperature resistance of polysiloxanes containing methyl or ethyl segments.

The DSC curves also show that compared with the silicone gel containing only Me_2_SiO segments (Entry 5A in Table 5), the *T*g values of other silicone gel samples containing Et_2_SiO segments are reduced. As the ethyl content in the silicone gel samples increases, their *T*g values exhibit a decreasing trend, indicating that the introduction of Et_2_SiO segments can reduce the *T*g of the polymer. Compared with the low-temperature crystallization phenomenon of PDES reported in the literature [18,48], changing the end-capping group or introducing Me_2_SiO segments or MeHSiO segments into the PDES segments inhibits the low-temperature crystallization behavior of PDES.

##### TGA and DTG Curves

The TGA and DTG curves of each silicone gel sample in the nitrogen atmosphere are shown in Figure 3c,d. The temperature at 5% weight loss (*T*_5%_) for each sample is determined from the TGA curve and is presented in Table 5. As can be seen from Figure 3c,d, the silicone gel sample composed of only Me_2_SiO segments (Entry 5A in Table 5) has a prominent thermal weight loss at 357 °C and 437 °C, and its thermal degradation rates are −0.22 wt%/°C and −0.25 wt%/°C, respectively. Other silicone gel samples containing Et_2_SiO segments (Entries 5B–5E in Table 5) showed three prominent thermal degradation stages.

As the ethyl content in the silicone gel increases, the first thermal degradation peak appears at 367~396 °C, and the thermal degradation rate ranges from −0.15 to −0.084 wt%/°C. The second thermal degradation peak appears at 455~478 °C, and its thermal degradation rate is between −0.19~−0.16 wt%/°C. The third thermal degradation peak appears at 565~579 °C, and its thermal degradation rate is between −1.02~−0.75 wt%/°C. The first thermal degradation process of each sample may be related to the dehydrogenation coupling of excessive Si-H remaining in the silicone gel, and the second thermal degradation process may be due to the cleavage of Si-CH_3_ at high temperatures to generate volatile CH_4_ and other small alkanes [49]. It may also be caused by the breakage of Si-O bonds caused by free radicals generated at high temperatures to produce volatile cyclosiloxanes. The third thermal degradation process may be related to the Et_2_SiO segment, because the thermal degradation rate of each silicone gel increases with the increase of ethyl content. It can be speculated that the third thermal degradation process may result from the chain scission of the polymer chain consisting of Et_2_SiO segments under the action of free radicals to generate a volatile cyclic Et_2_SiO mixture, resulting in a significant weight loss in the silicone gel between 565 and 579 °C. Although the third thermal degradation temperature of each silicone gel sample containing Et_2_SiO segments is higher than those of the other two stages, the thermal degradation rate is significantly accelerated, resulting in the residual weight of these silicone gel samples at 800 °C being significantly lower than that of a silicone gel sample containing no Et_2_SiO segments (Entry 5A in Table 5). The larger mass loss in these systems may also be caused by the larger percentage of the organic fraction subjected to volatilization, since the ethyl substituent group is larger than the methyl substituent group. It can be seen that although the introduction of Et_2_SiO segments into polysiloxane molecules can reduce the *T*g of silicone gel, the high-temperature resistance of the resulting polymer material is reduced.

### 3.5. Possible Catalytic Mechanism

**Scheme 3:**
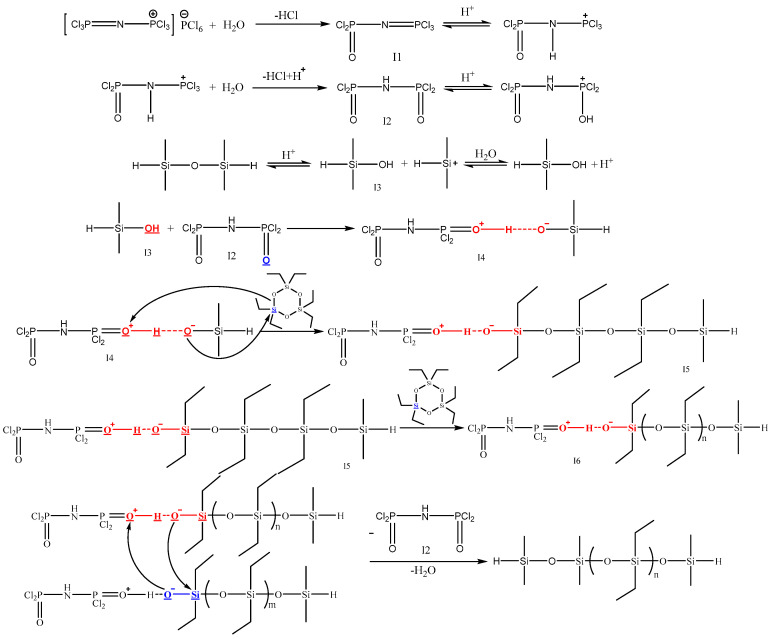
Reaction mechanism diagram for the preparation of siloxane oligomers or copolymers by linear chlorinated phosphazene acid-catalyzed ring-opening polymerization of cyclosiloxanes.

The reaction of the linear chlorinated phosphazene acid-catalyzed ring-opening polymerization of cyclosiloxanes to prepare siloxane oligomers or copolymers is essentially a cation-catalyzed ring-opening polymerization of cyclosiloxanes. Taking the cationic ring-opening polymerization reaction of D_3_^Et^ and M^H^M^H^ as an example, the possible reaction mechanism is speculated to proceed as shown in Scheme 3 [50]. First, the linear chlorinated phosphazene acid interacts with the residual water molecules in the system, and the P-Cl bond cleaves to generate the P=O bond to obtain intermediate I1. Intermediate I1, which is protonated due to the acidic conditions, interacts with water molecules again to form intermediate I2. At the same time, the end-capping agent M^H^M^H^ is cleaved into intermediate I3 in an acidic environment and interacts with intermediate I2 to generate intermediate I4. Intermediate I4 interacts with D_3_^Et^ to open the ring to obtain intermediate I5. Subsequent chain growth was brought about using the same principle, which resulted in intermediate I6. The two intermediates I6 interact to remove water molecules and intermediate I2 to obtain the final desired product.

## 4. Conclusions

Linear chlorinated phosphazene acid can catalyze the equilibrium polymerization of cyclosiloxanes under mild conditions. This is especially true of the equilibrium copolymerization of stable D_3_^Et^ with D_4_^H^, D_4_, and M^H^M^H^ to prepare PDES oligomers or their copolymers containing active Si-H bonds, which overcomes the disadvantage of current catalytic systems, which cannot prepare PDES oligomers or copolymers containing active Si-H bonds either in the terminal or side chain of the polymer. The prepared polymer containing Si-H bonds and Et_2_SiO segments has a narrow molecular weight distribution and a clear and transparent liquid appearance.

When the linear chlorinated phosphazene acid is used as the catalyst, M^Vi^M^Vi^ functions as the end-capping agent, and D_3_^Et^ is used as the monomer, polymers containing active Si-CH=CH_2_ bonds and Et_2_SiO segments can be prepared. Compared with KOH, the polymerization reaction catalyzed by a phosphazene acid catalyst can be more efficiently conducted under lower temperatures and with a minor dosage of catalyst. Furthermore, the reaction process is simple, controllable, and offers a higher yield.

A series of silicone gels containing Et_2_SiO segments was prepared using the hydrosilylation reaction of the active Si-H and Si-CH=CH_2_ functionalized PDES oligomers or copolymers prepared in this study. The gels were then compared with gels consisting of pure Me_2_SiO segments. It was found that the glass transition temperature of the silicone gel decreased when an Et_2_SiO segment was introduced, and the thermal properties of the silicone gel decreased with the increase of its ethyl content. No apparent crystallization or melting behavior was observed in the prepared silicone gel within the investigation range, and it has a wide service temperature range.

## Figures and Tables

**Table 1:** Preparation of *α*, *ω*-bisdimethylsiloxyl-terminated PDES oligomers (PDES-H).

Entry	*m*_MH_^a^/g	*m*_D3Et_^b^/g	*m*_Cat_^c^/g	*C*_Cat_^d^/ppm	*RT*/°C	*t*/h	*m*_exp_	*η*_25_/(mPa.s)	*M*_NMR_/(g·mol^−1^)	*M*n/(g·mol^−1^)	PDI	*n*_D_^25^	Yield/%
1A	3.13	21.46	0.4918	500	50	5	11	39.50	1256	900	1.46	1.4370	50
1B	3.13	21.46	0.4918	500	50	7	12	41.00	1358	840	1.50	1.4371	93
1C	3.00	20.54	0.4708	500	50	9	11	43.00	1256	900	1.46	1.4379	93
1D	3.13	21.46	0.4918	500	50	11	11	45.00	1256	760	1.54	1.4371	86
1E	3.09	21.16	0.1940	200	60	9	13	46.00	1460	1160	1.66	1.4389	50
1F	3.09	21.16	0.2910	300	60	9	12	47.75	1358	800	1.42	1.4378	88
1G	3.09	21.16	0.3880	400	60	9	12	44.50	1358	750	1.41	1.4371	66
1H	3.09	21.16	0.4850	500	60	9	14	48.75	1562	920	1.69	1.4378	78
1I	3.09	21.16	0.4850	500	70	9	13	42.75	1460	850	1.66	1.4371	85
1J	3.09	21.16	0.4850	500	90	9	13	31.75	1562	820	1.63	1.4370	90

Note: a. M^H^M^H^; b. D_3_^Et^; c. The mass of catalyst with a mass fraction of 2.5 wt% in DCM; d. The relative dosage of the catalyst based on the sum of the mass of M^H^M^H^ and D_3_^Et^. The designed degree of polymerization is *m* = 9. HDMS is used as the passivator, and its dosage is 7.5 times the catalyst dosage.

**Table 2:** Experimental results of *α*, *ω*-bisdimethylsiloxyl-terminated PMHS-*co*-PDES.

Entry	*m*_MH_^a^/g	*m*_D3Et_^b^/g	*m*_D4H_^c^/g	*m*_Cat_^d^/g	*C*_Cat_^e^/ppm	*RT*/℃	*m*_exp_	*n*_exp_	*M*_NMR_/(g.mol^−1^)	*η*_25_/(mPa.s)	*M*n/(g.mol^−1^)	PDI	*n*_D_^25^	Yield/%
2A	2.42	16.56	8.66	0.5528	500	50	7.4	5.2	1201	42.00	3000	1.98	1.4248	58
2B	2.42	16.56	8.66	0.5528	500	60	7.3	5.4	1203	26.00	1800	1.77	1.4231	68
2C	2.42	16.56	8.66	0.4422	400	60	10.0	5.0	1454	30.25	2200	1.81	1.4231	69
2D	2.42	16.56	8.66	0.3317	300	60	8.3	5.3	1300	34.00	2300	1.98	1.4239	61
2E	2.42	16.56	8.66	0.2211	200	60	6.2	5.3	1084	27.25	1800	1.71	1.4232	37

Note: a. M^H^M^H^; b. D_3_^Et^; c. D_4_^H^; d. The mass of catalyst with a mass fraction of 2.5 wt% in DCM; e. the relative dosage of the catalyst based on the sum of the mass of M^H^M^H^, D_3_^Et^ and D_4_^H^. The degree of polymerization of Et_2_SiO segments and MeHSiO segments are designed to be *m* = 9 and *n* = 8, respectively. HDMS is used as the passivator, and its dosage is 7.5 times that of the catalyst. The reaction time is 8 h.

**Table 3:** Experimental results of *α*, *ω-*bistrimethylsiloxyl-terminated PMHS-*co*-PDES.

Entry	*m*_MM_^a^/g	*m*_D3Et_^b^/g	*m*_D4H_^c^/g	*m*_Cat_^d^/g	*C*_Cat_^e^/ppm	*m*_exp_	*n*_exp_	*M*_NMR_/(g.mol^−1^)	*η*_25_/(mPa.s)	*M*n/(g.mol^−1^)	PDI	*n*_D_^25^	Yield/%
3A	3.00	16.43	8.59	0.5604	500	13.2	8.1	1994	38.75	2200	2.30	1.4241	81
3B	2.52	13.80	7.22	0.3766	400	6.8	7.5	1306	30.25	1700	1.94	1.4233	69
3C	2.52	13.80	7.22	0.2825	300	7.8	7.6	1414	29.25	1700	1.87	1.4230	68
3D	2.52	13.80	7.22	0.1883	200	7.9	8.1	1454	36.00	2100	1.92	1.4238	61

Note: a. MM; b. D_3_^Et^; c. D_4_^H^; d. The mass of catalyst with a mass fraction of 2.5 wt% in DCM; e. the relative dosage of the catalyst based on the sum of the mass of MM, D_3_^Et^, and D_4_^H^. The degree of polymerization of Et_2_SiO segments and MeHSiO segments are designed to be *m* = 9 and *n* = 8, respectively. HDMS is used as the passivator, and its dosage is 7.5 times that of the catalyst. The reaction time is 8 h and the reaction temperature is 90 °C.

**Table 4:** *α*, *ω-*bisdimethylsiloxyl-terminated PDMS-*co*-PDES.

Entry	*m*_MH_^a^/g	*m*_D3Et_^b^/g	*m*_D4_^c^/g	*m*_Cat_^d^/g	*C*_Cat_^e^/ppm	*T*/℃	*m*_exp_	*n*_exp_	*M*_NMR_/(g.mol^−1^)	*η*_25_/(mPa.s)	*M*n/(g.mol^−1^)	PDI	*n*_D_^25^	Yield/%
4A	2.42	16.56	10.68	0.5932	500	50	20.0	18.0	3506	169.4	3900	1.88	1.4278	83
4B	2.42	16.56	10.68	0.5932	500	60	12.8	11.8	2313	57.75	1730	1.93	1.4258	69
4C	2.42	16.56	10.68	0.4746	400	60	13.0	12.0	2348	57.00	1660	1.93	1.4269	67
4D	2.42	16.56	10.68	0.3559	300	60	12.0	11.0	2172	57.00	1670	1.90	1.4270	69
4E	2.42	16.56	10.68	0.2373	200	60	12.5	12.0	2297	59.75	1700	1.87	1.4271	65

Note: a. M^H^M^H^; b. D_3_^Et^; c. D_4_; d. The mass of catalyst with a mass fraction of 2.5 wt% in DCM; e. the relative dosage of the catalyst based on the sum of the mass of M^H^M^H^, D_3_^Et^, and D_4_. The degree of polymerization of Et_2_SiO segments and Me_2_SiO segments are designed to be *m* = 9 and *n* = 8, respectively. HDMS is used as the passivator, and its dosage is 7.5 times that of the catalyst. The reaction time is 8 h.

**Table 5:** Compositions of silicone gel samples and their thermal properties.

Entry	Si-Vi Polymer^a^	Structure	*m*^b^/g	Si-H Polymer^c^	Structure	*m*^d^/g	*w*_(Si-Et)_^e^/%	*Tg*^f^/°C	*Tg*^g^/°C	*T*_5%_^h^/°C
5A	PDMS-273	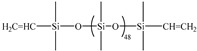	2.00	UC-203-29	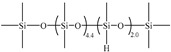	0.5167	0	−124.5	−122.1	347
5B	PDES-Vi	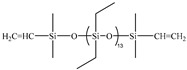	2.00	UC-203-29	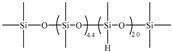	1.2771	30.43	−131.9	−124.2	341
5C	PDES-Vi	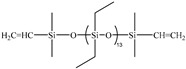	2.00	UC-613-47	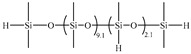	0.7881	35.77	−132.9	−125.3	326
5D	PDES-Vi	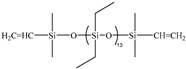	2.00	Entry 2B	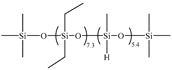	0.6382	44.87	−134.3	−128.8	297
5E	PDES-Vi	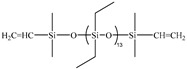	2.00	Entry 3B	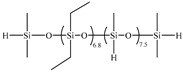	0.6024	46.44	−138.6	−126.9	312

Note: a. polysiloxanes containing Si-CH=CH_2_ bonds; b. mass of polysiloxane samples containing Si-CH=CH_2_ bonds; c. polysiloxanes containing Si-H bonds; d. mass of polysiloxane samples containing Si-H bonds; e. content of ethyl group measured by area integration from ^1^H NMR spectra; f. glass transition temperature of silicone gel samples measured in temperature decreasing stage; g. glass transition temperature of silicone gel samples measured in temperature increasing stage; h. temperature at 5% weight loss. Using platinum-vinyl complex (Karstedt’s catalyst) with a Pt content of 0.1 wt% as the catalyst, all silicone gels were cured at 80 °C for 1 h.

## Data Availability

Structure characterizations of the prepared PDES oligomers, PMHS-*co*-PDES, and PDMS-*co*-PDES samples with other molecular weights can be found in the supplementary information of this manuscript.

## Supplementary Materials

The following supporting information can be downloaded at: https://www.mdpi.com/article/10.3390/polym16192835/s1, Figure S1: NMR spectra of PDES-H fluid using CDCl_3_ with trace amount of TMS as solvent (Entry 1C in Table 1. a. ^1^H NMR spectrum; b. ^13^C NMR spectrum; c. ^29^Si NMR spectrum.); Figure S2: NMR and FT-IR spectra of PDES-Vi fluid using CDCl_3_ with trace amount of TMS as solvent (a. ^1^H NMR spectrum; b. ^13^C NMR spectrum; c. ^29^Si NMR spectrum; d. FT-IR spectrum.); Figure S3: NMR spectra of *α*, *ω*-bisdimethylsiloxyl-terminated PMHS-*co*-PDES using CDCl_3_ with trace amount of TMS as solvent (Entry 2A in Table 2. a. ^1^H NMR spectrum of the mixture of 9.7 mg sample, 2.8 mg 1,4-dioxane and CDCl_3_; b. ^13^C NMR spectrum; c. ^29^Si NMR spectrum.); Figure S4: NMR spectra and GPC curve of *α*, *ω*-bisdimethylsiloxyl-terminated PMHS-*co*-PDES using CDCl_3_ with trace amount of TMS as solvent (Entry 2B in Table 2. a. ^1^H NMR spectrum of the mixture of 13.8 mg sample, 4.2 mg 1,4-dioxane and CDCl_3_; b. ^13^C NMR spectrum; c. GPC curve.); Figure S5: NMR spectra and GPC curve of *α*, *ω*-bisdimethylsiloxyl-terminated PMHS-*co*-PDES using CDCl_3_ with trace amount of TMS as solvent (Entry 2C in Table 2. a. ^1^H NMR spectrum of the mixture of 9.9 mg sample, 5.9 mg 1,4-dioxane and CDCl_3_; b. ^13^C NMR spectrum; c. GPC curve.); Figure S6: NMR spectra and GPC curve of *α*, *ω*-bisdimethylsiloxyl-terminated PMHS-*co*-PDES using CDCl_3_ with trace amount of TMS as solvent (Entry 2D in Table 2. a. ^1^H NMR spectrum of the mixture of 13.9 mg sample, 4.2 mg 1,4-dioxane and CDCl_3_; b. ^13^C NMR spectrum; c. GPC curve.); Figure S7: NMR spectra and GPC curve of *α*, *ω*-bisdimethylsiloxyl-terminated PMHS-*co*-PDES using CDCl_3_ with trace amount of TMS as solvent (Entry 2E in Table 2. a. ^1^H NMR spectrum of the mixture of 10.4 mg sample, 5.8 mg 1,4-dioxane and CDCl_3_; b. ^13^C NMR spectrum; c. GPC curve.); Figure S8: NMR spectra of *α*, *ω*-bistrimethylsiloxyl-terminated PMHS-*co*-PDES using CDCl_3_ with trace amount of TMS as solvent (Entry 3A in Table 3. a. ^1^H NMR spectrum of the mixture of 9.5 mg sample, 2.9 mg 1,4-dioxane and CDCl_3_; b. ^13^C NMR spectrum; c. ^29^Si NMR spectrum.); Figure S9: NMR spectra and GPC curve of *α*, *ω*-bistrimethylsiloxyl-terminated PMHS-*co*-PDES using CDCl_3_ with trace amount of TMS as solvent (Entry 3B in Table 3. a. ^1^H NMR spectrum of the mixture of 13.5 mg sample, 5.3 mg 1,4-dioxane and CDCl_3_; b. ^13^C NMR spectrum; c. GPC curve.); Figure S10: NMR spectra and GPC curve of *α*, *ω*-bistrimethylsiloxyl-terminated PMHS-*co*-PDES using CDCl_3_ with trace amount of TMS as solvent (Entry 3C in Table 3. a. ^1^H NMR spectrum of the mixture of 11.2 mg sample, 4.1 mg 1,4-dioxane and CDCl_3_; b. ^13^C NMR spectrum; c. GPC curve.); Figure S11: NMR spectra and GPC curve of *α*, *ω*-bistrimethylsiloxyl-terminated PMHS-*co*-PDES using CDCl_3_ with trace amount of TMS as solvent (Entry 3D in Table 3. a. ^1^H NMR spectrum of the mixture of 11.5 mg sample, 5.8 mg 1,4-dioxane and CDCl_3_; b. ^13^C NMR spectrum; c. GPC curve.); Figure S12: NMR spectra of *α*, *ω*-bisdimethylsiloxyl-terminated PDMS-*co*-PDES using CDCl_3_ with trace amount of TMS as solvent (Entry 4A in Table 4. a. ^1^H NMR spectrum; b. ^13^C NMR spectrum; c. ^29^Si NMR spectrum.); Figure S13: NMR spectra and GPC curve of *α*, *ω*-bisdimethylsiloxyl-terminated PDMS-*co*-PDES using CDCl_3_ with trace amount of TMS as solvent (Entry 4B in Table 4. a. ^1^H NMR spectrum; b. ^13^C NMR spectrum; c. GPC curve.); Figure S14: NMR spectra and GPC curve of *α*, *ω*-bisdimethylsiloxyl-terminated PDMS-*co*-PDES using CDCl_3_ with trace amount of TMS as solvent (Entry 4C in Table 4. a. ^1^H NMR spectrum; b. ^13^C NMR spectrum; c. GPC curve.); Figure S15: NMR spectra and GPC curve of *α*, *ω*-bisdimethylsiloxyl-terminated PDMS-*co*-PDES using CDCl_3_ with trace amount of TMS as solvent (Entry 4D in Table 4. a. ^1^H NMR spectrum; b. ^13^C NMR spectrum; c. GPC curve.); Figure S16: NMR spectra and GPC curve of *α*, *ω*-bisdimethylsiloxyl-terminated PDMS-*co*-PDES using CDCl_3_ with trace amount of TMS as solvent (Entry 4E in Table 4. a. ^1^H NMR spectrum; b. ^13^C NMR spectrum; c. GPC curve.). The calculation method of molecular weight (*M*_NMR_) and degree of polymerization for PDES oligomers or copolymers from ^1^H NMR spectrum are provided Section Part II of the Supplementary Materials.
