# Supplementary material for: Polymers Containing Diethylsiloxane Segment and Active Functional Group by Ring-Opening Polymerization of Hexaethylcyclotrisiloxane under the Catalysis of Linear Chlorinated Phosphazene Acid
Source: Polymers (Basel). 2024 Oct 7;16(19):2835. doi: 10.3390/polym16192835 (PMC11478888; doi:10.3390/polym16192835)
Supplement: Supplementary file 1 [file polymers-16-02835-s001.zip › polymers-3204403-supplementary.pdf]

Polymers Containing Diethylsiloxane Segment and Active Functional Group by Ring-Opening Polymerization of Hexaethylcyclotrisiloxane Under the Catalysis of Linear Chlorinated Phosphazene Acid

Chen Jin, Hao Yang, Yang Zhang, Shuting Zhang, Xu Long, Hong Dong, Yanjiang Song, Zhirong Qu, Chuan Wu*

College of Material, Chemistry and Chemical Engineering, Key Laboratory of Organosilicon Chemistry and Material Technology, Ministry of Education, Hangzhou Normal University, Hangzhou, 311121, Zhejiang, People's Republic of China

Supporting Information

Part I. ^{1}H NMR, ^{13}C NMR, ^{29}Si NMR spectra, GPC curves, TGA and DTG curves of synthesized polymers

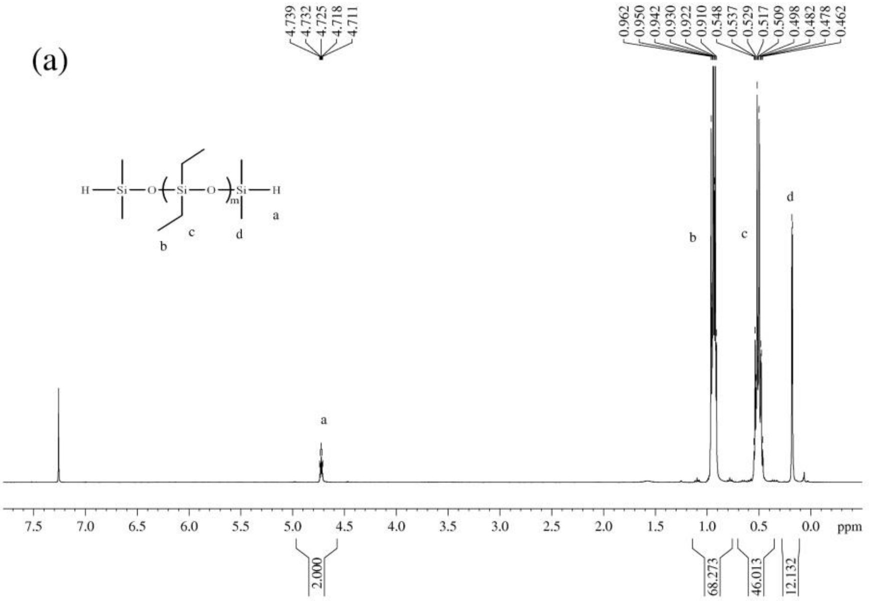

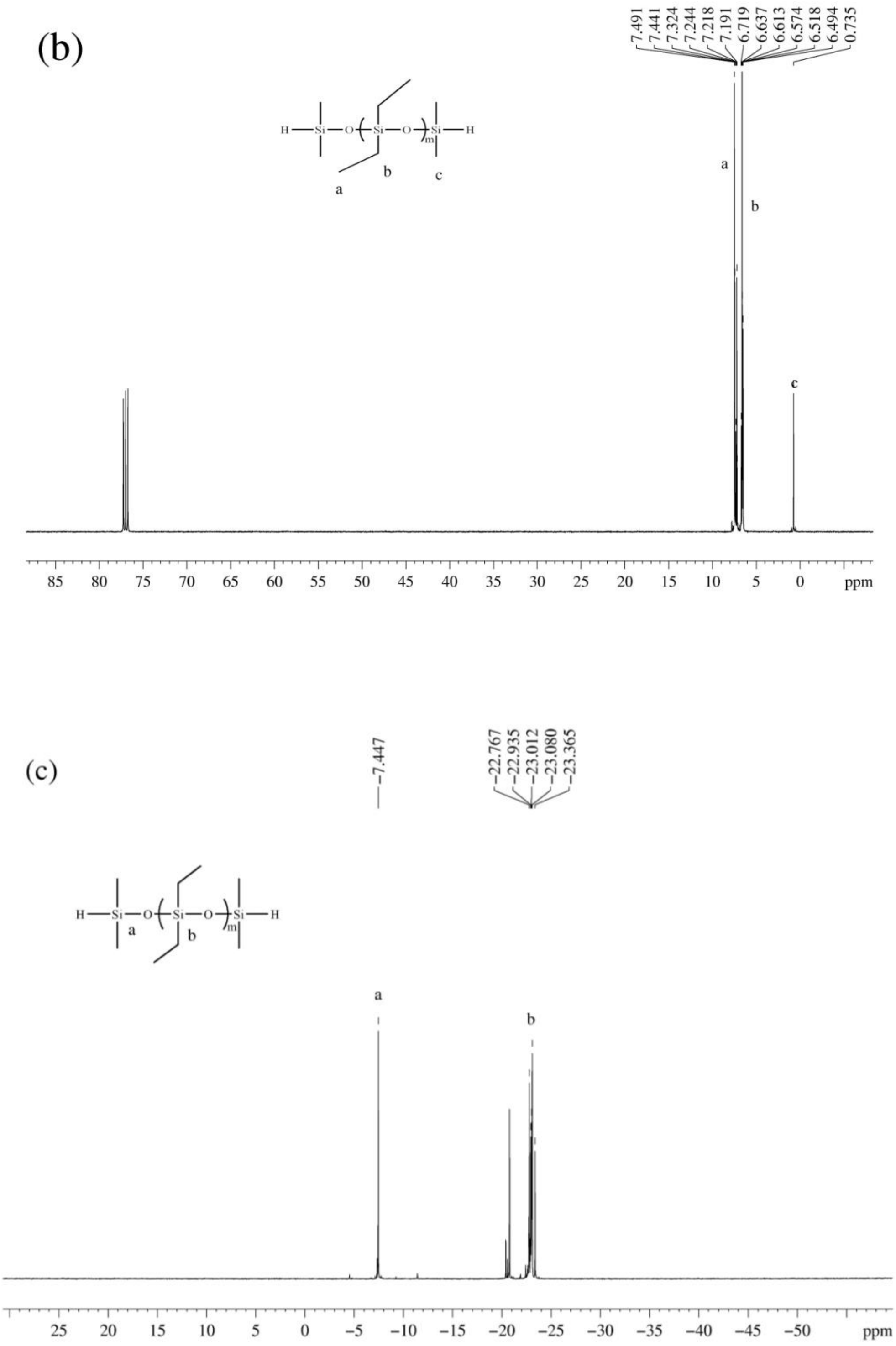

Figure S1. NMR spectra of PDES-H fluid using $CDCl_3$ with trace amount of TMS as solvent (Entry 1C in Table 1.

a. ^{1}H NMR spectrum; b. ^{13}C NMR spectrum; c. ^{29}Si NMR spectrum.)

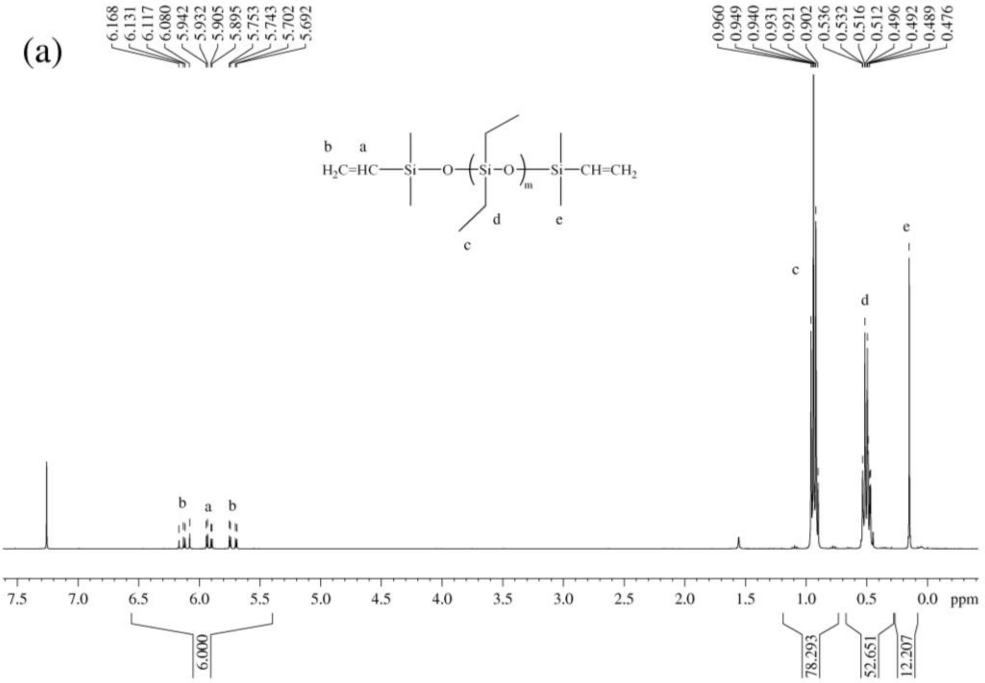
(a)
6.168
6.131
6.117
6.080
5.942
5.932
5.905
5.895
5.753
5.743
5.702
5.692
0.960
0.949
0.940
0.931
0.921
0.902
0.536
0.532
0.516
0.512
0.496
0.492
0.489
0.476
b a
H_2C=HC—Si—O—(Si—O)m—Si—CH=CH_2
d e
c
6.000
78.293
52.651
12.207
ppm

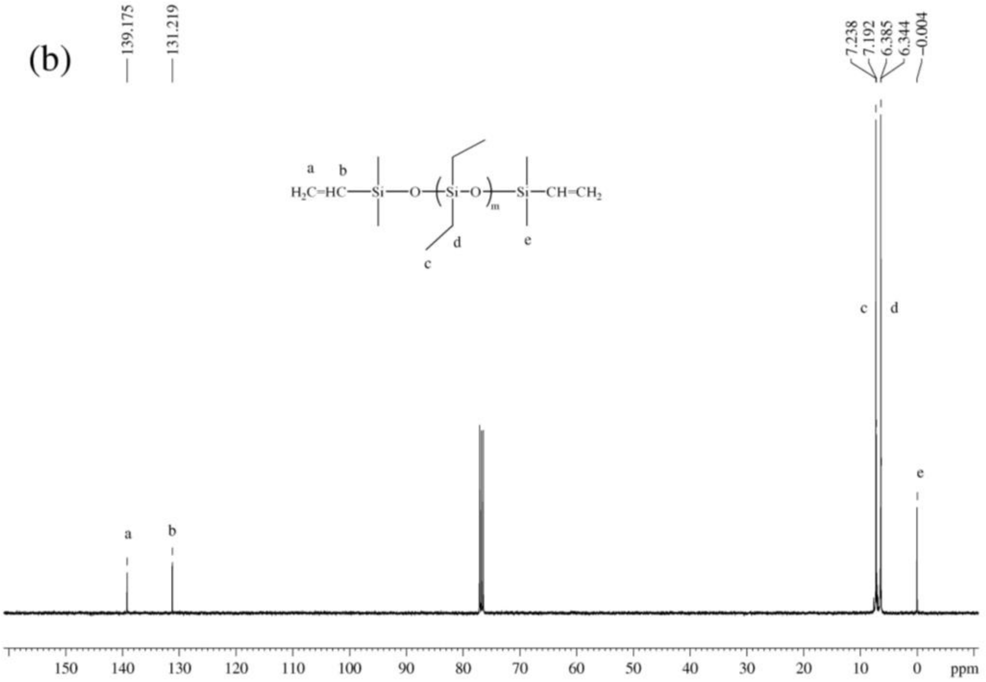
(b)
139.175
131.219
7.238
7.192
6.385
6.344
-0.004
a b
H_2C=HC—Si—O—(Si—O)m—Si—CH=CH_2
d e
c
ppm

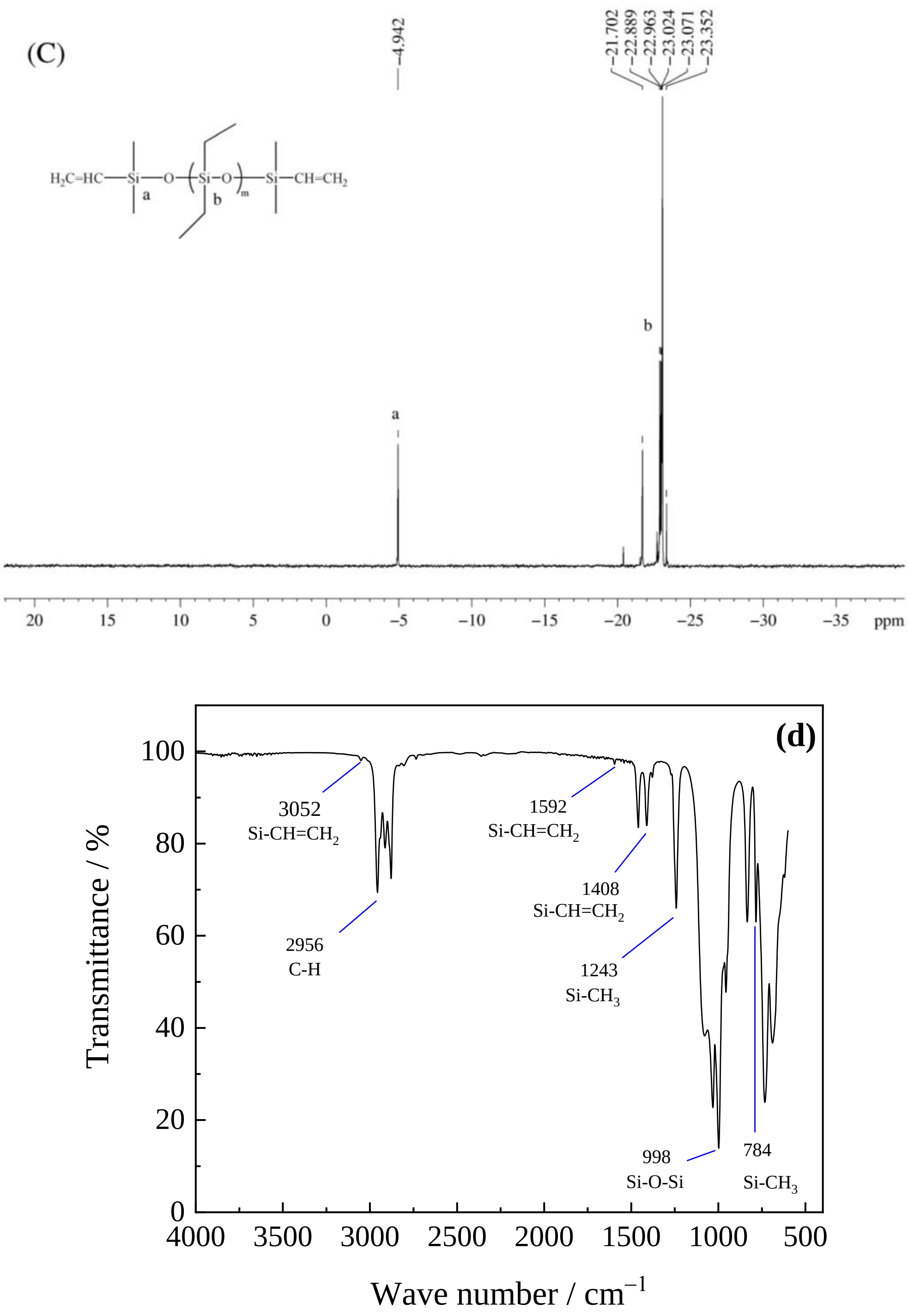

Figure S2. NMR and FT-IR spectra of PDES-Vi fluid using $CDCl_3$ with trace amount of TMS as solvent (a. ^{1}H NMR spectrum; b. ^{13}C NMR spectrum; c. ^{29}Si NMR spectrum; d. FT-IR spectrum.)

(a)

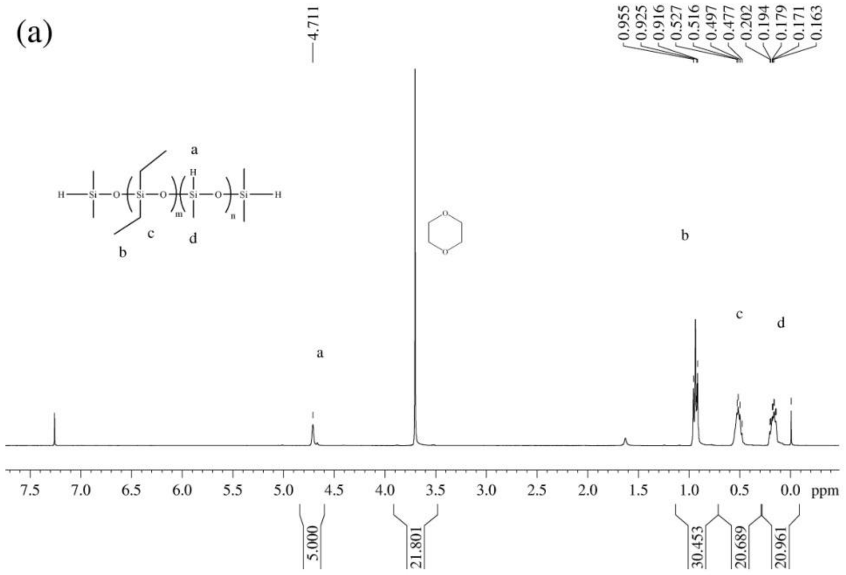

4.711
0.955
0.925
0.916
0.527
0.516
0.497
0.477
0.202
0.194
0.179
0.171
0.163
a
b
c
d
7.5 7.0 6.5 6.0 5.5 5.0 4.5 4.0 3.5 3.0 2.5 2.0 1.5 1.0 0.5 0.0 ppm
5.000
21.801
30.453
20.689
20.961

(b)

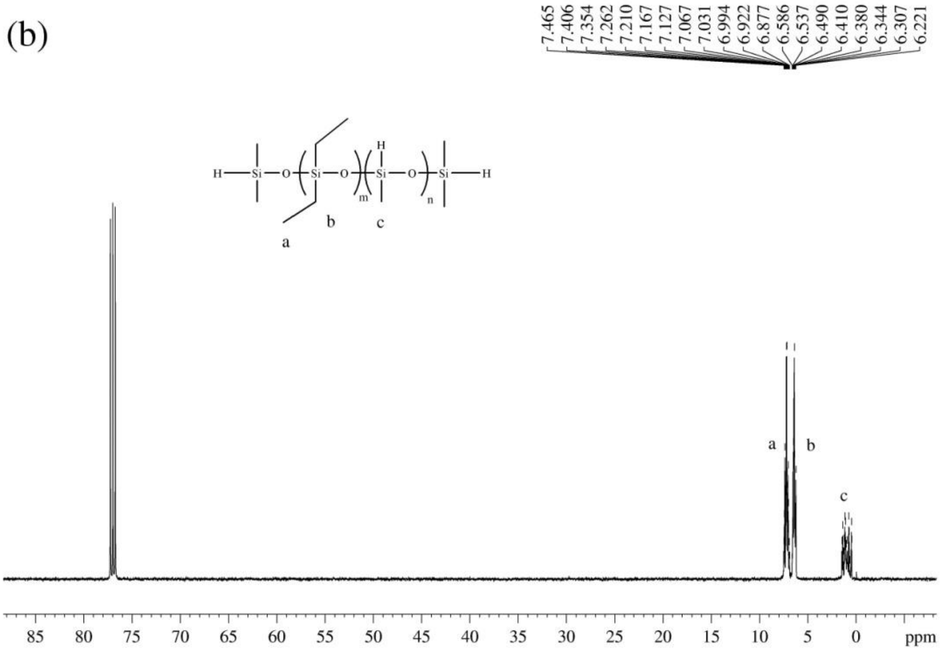

7.465
7.406
7.354
7.262
7.210
7.167
7.127
7.067
7.031
6.994
6.922
6.877
6.586
6.537
6.490
6.410
6.380
6.344
6.307
6.221
a
b
c
85 80 75 70 65 60 55 50 45 40 35 30 25 20 15 10 5 0 ppm

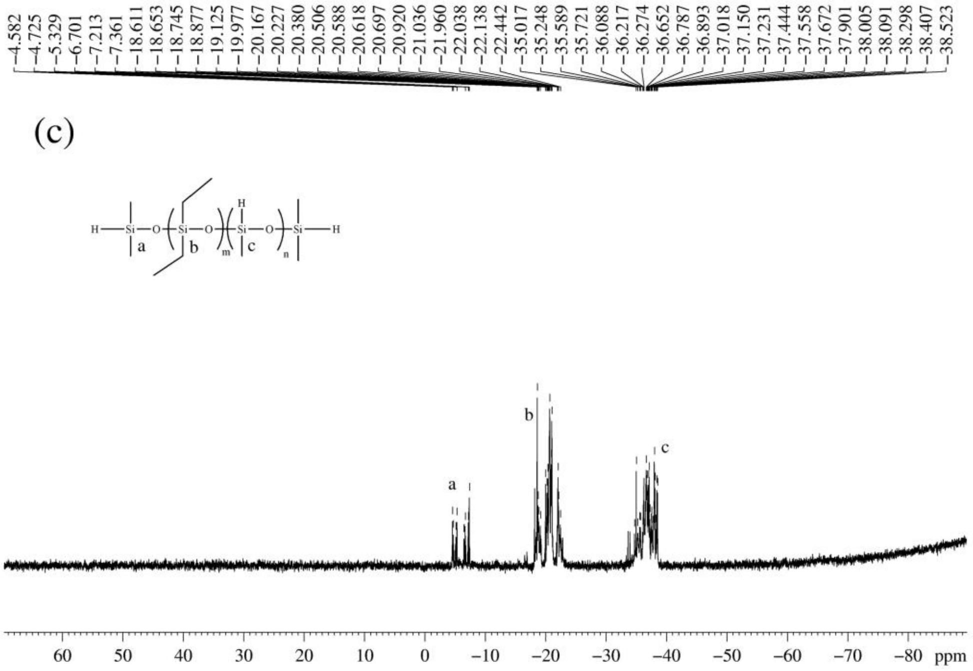

Figure S3. NMR spectra of α, ω-bisdimethylsiloxyl-terminated PMHS-*co*-PDES using $CDCl_3$ with trace amount of TMS as solvent (Entry 2A in Table 2. a. ^{1}H NMR spectrum of the mixture of 9.7 mg sample, 2.8 mg 1,4-dioxane and $CDCl_3$; b. ^{13}C NMR spectrum; c. ^{29}Si NMR spectrum.)

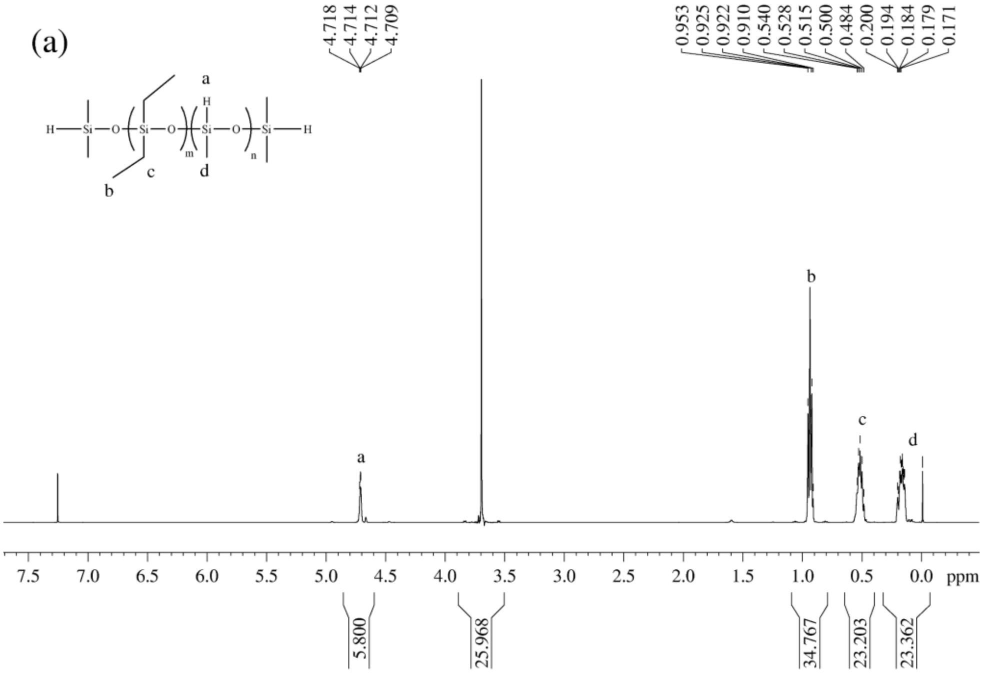

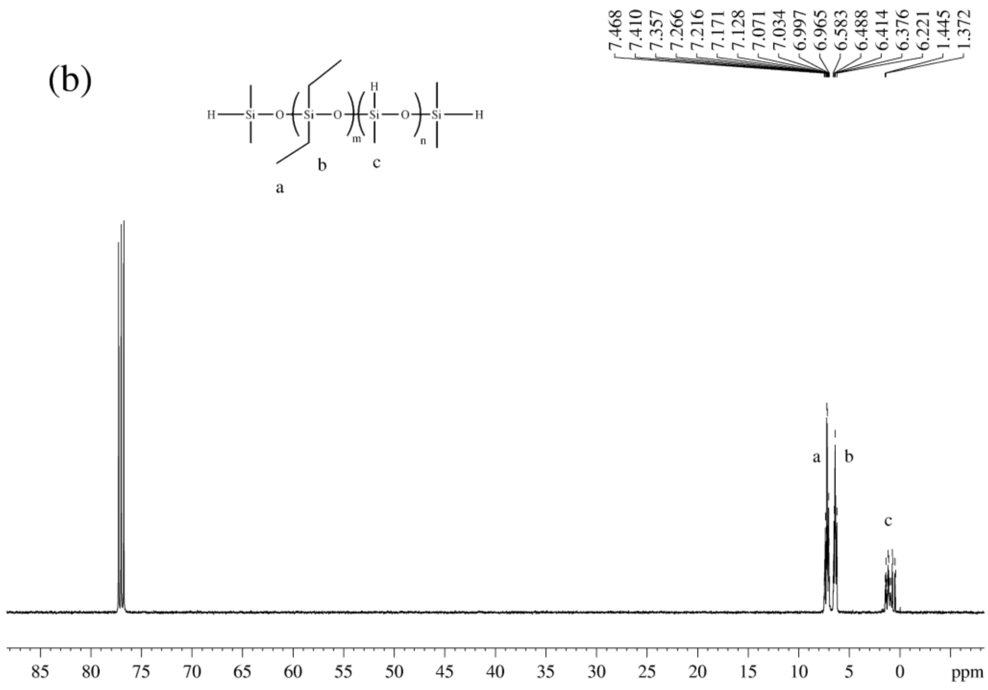

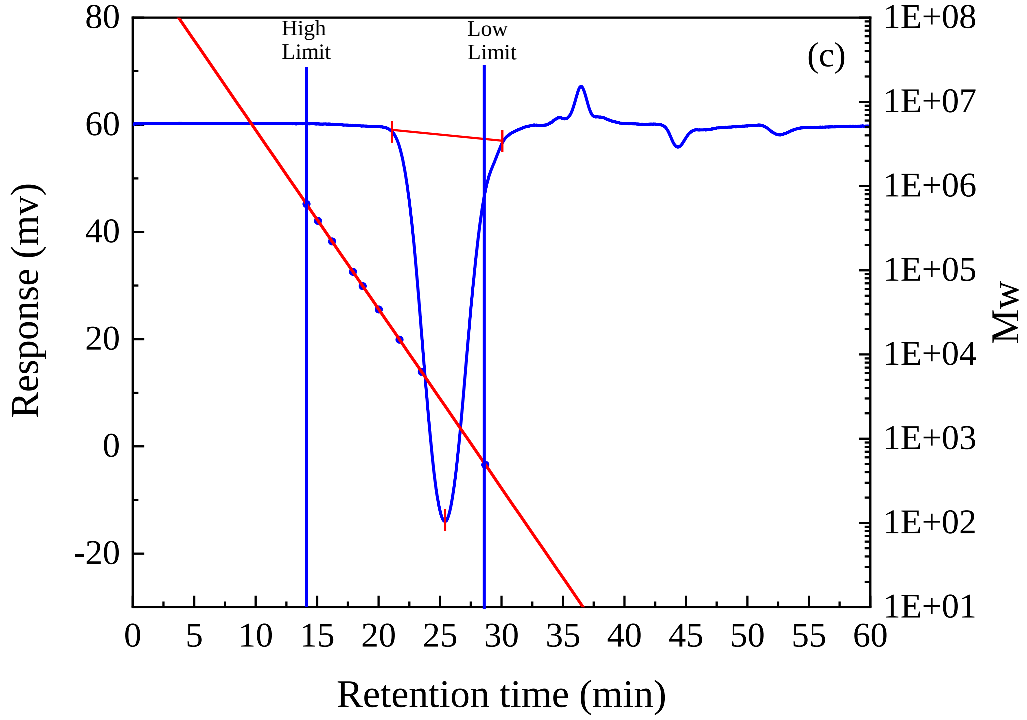

Figure S4. NMR spectra and GPC curve of α, ω-bisdimethylsiloxyl-terminated PMHS-*co*-PDES using $CDCl_3$ with trace amount of TMS as solvent (Entry 2B in Table 2. a. ^{1}H NMR spectrum of the mixture of 13.8 mg sample, 4.2 mg 1,4-dioxane and $CDCl_3$; b. ^{13}C NMR spectrum; c. GPC curve.)

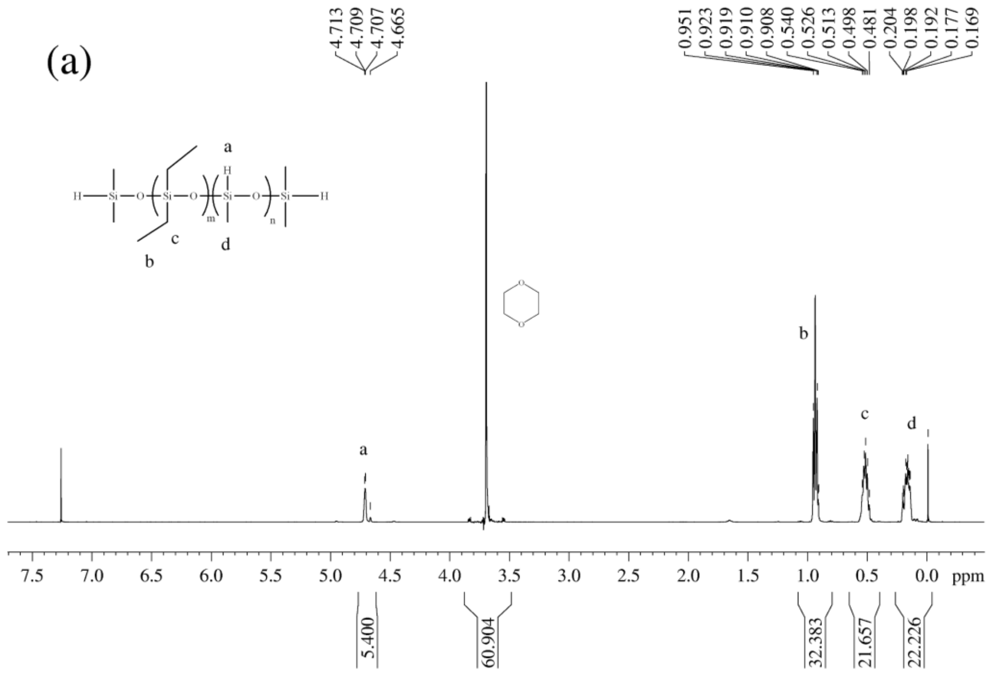
(a)
4.713
4.709
4.707
4.665
0.951
0.923
0.919
0.910
0.908
0.540
0.526
0.513
0.498
0.481
0.204
0.198
0.192
0.177
0.169
a
b
c
d
7.5 7.0 6.5 6.0 5.5 5.0 4.5 4.0 3.5 3.0 2.5 2.0 1.5 1.0 0.5 0.0 ppm
5.400
60.904
32.383
21.657
22.226

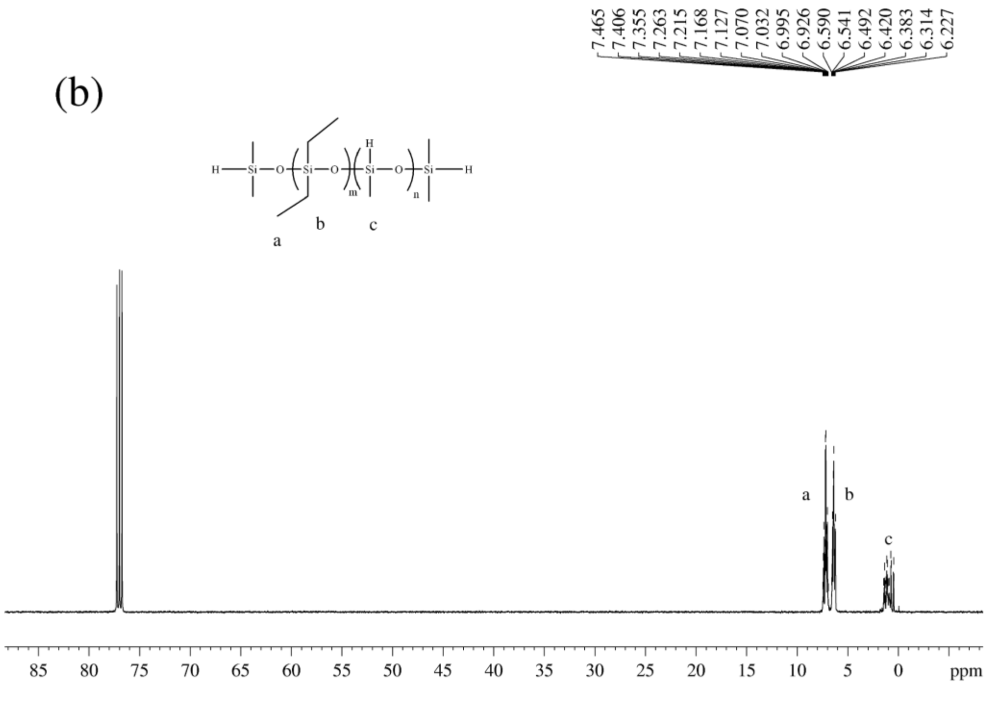
(b)
7.465
7.406
7.355
7.263
7.215
7.168
7.127
7.070
7.032
6.995
6.926
6.590
6.541
6.492
6.420
6.383
6.314
6.227
a
b
c
85 80 75 70 65 60 55 50 45 40 35 30 25 20 15 10 5 0 ppm

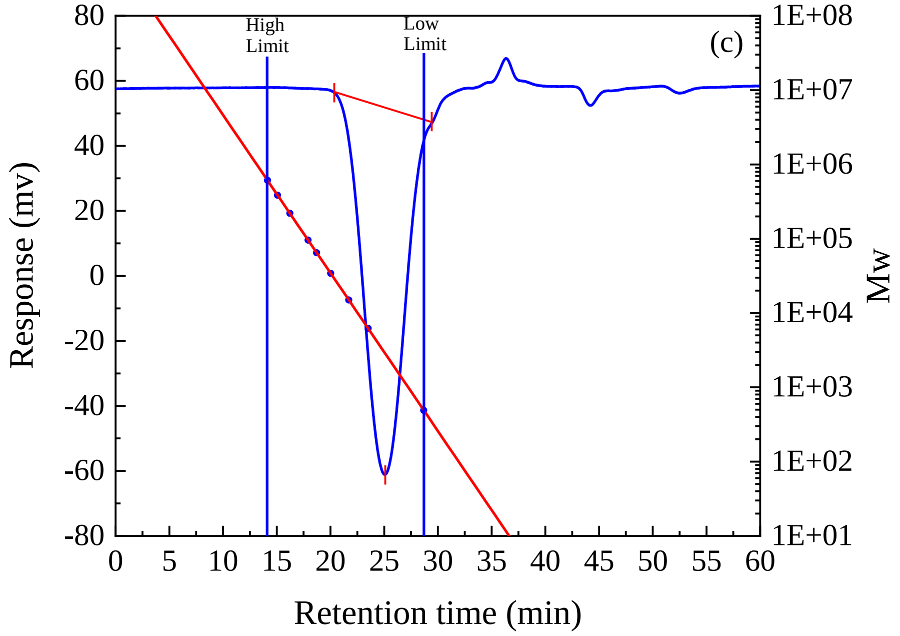

Figure S5. NMR spectra and GPC curve of *α*, *ω*-bisdimethylsiloxyl-terminated PMHS-*co*-PDES using $CDCl_3$ with trace amount of TMS as solvent (Entry 2C in Table 2. a. ^{1}H NMR spectrum of the mixture of 9.9 mg sample, 5.9 mg 1,4-dioxane and $CDCl_3$; b. ^{13}C NMR spectrum; c. GPC curve.)

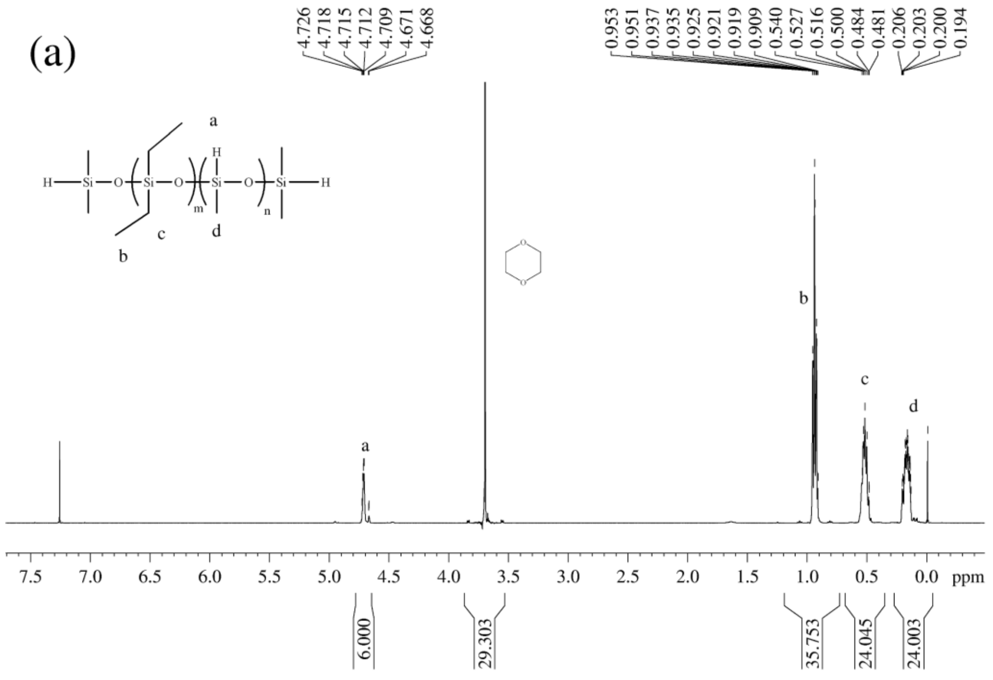

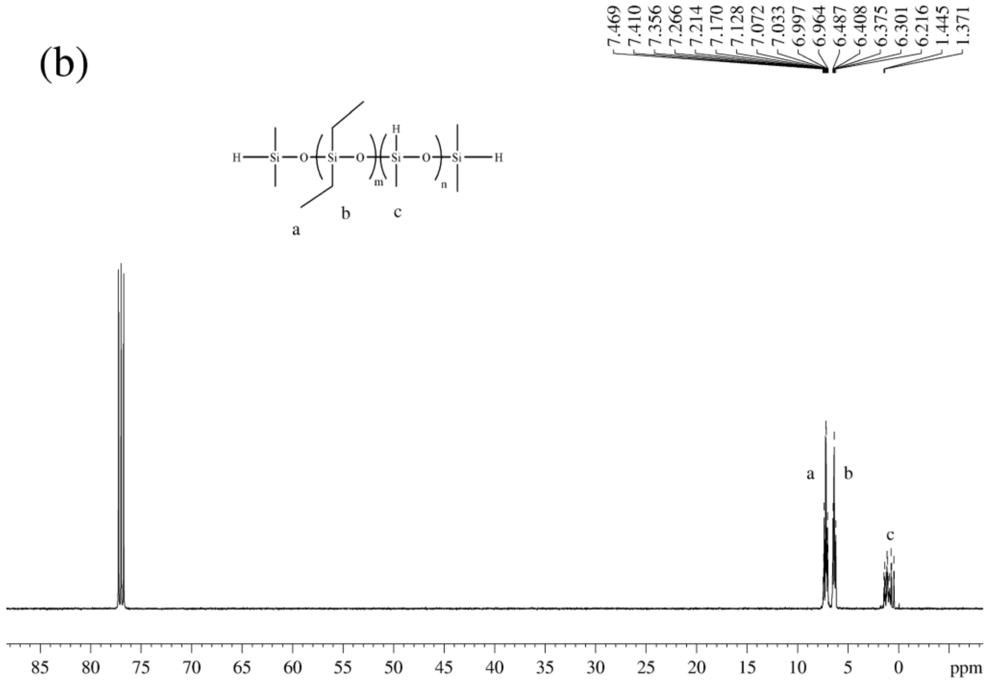

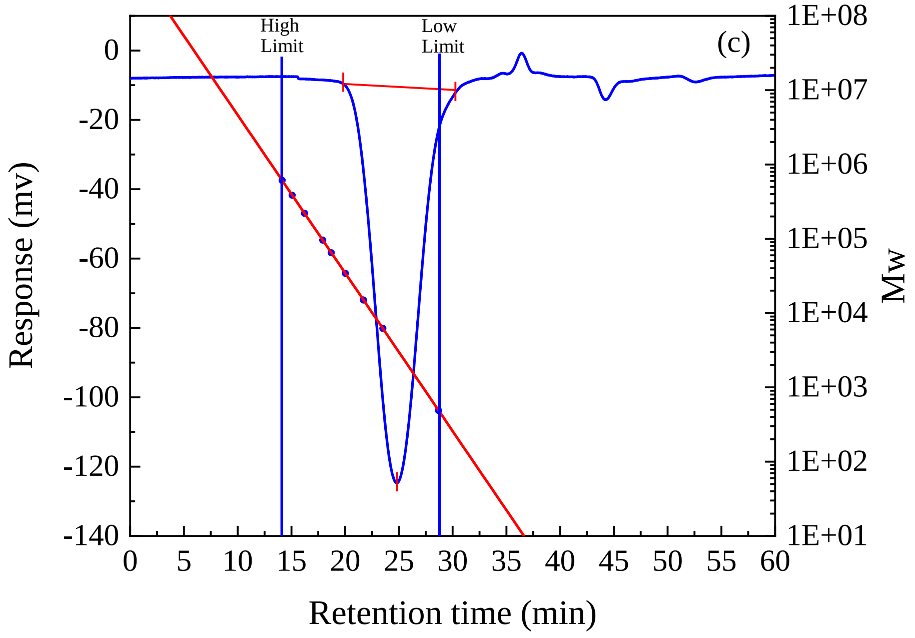

Figure S6. NMR spectra and GPC curve of α, ω-bisdimethylsiloxyl-terminated PMHS-*co*-PDES using $CDCl_3$ with trace amount of TMS as solvent (Entry 2D in Table 2. a. ^{1}H NMR spectrum of the mixture of 13.9 mg sample, 4.2 mg 1,4-dioxane and $CDCl_3$; b. ^{13}C NMR spectrum; c. GPC curve.)

(a)

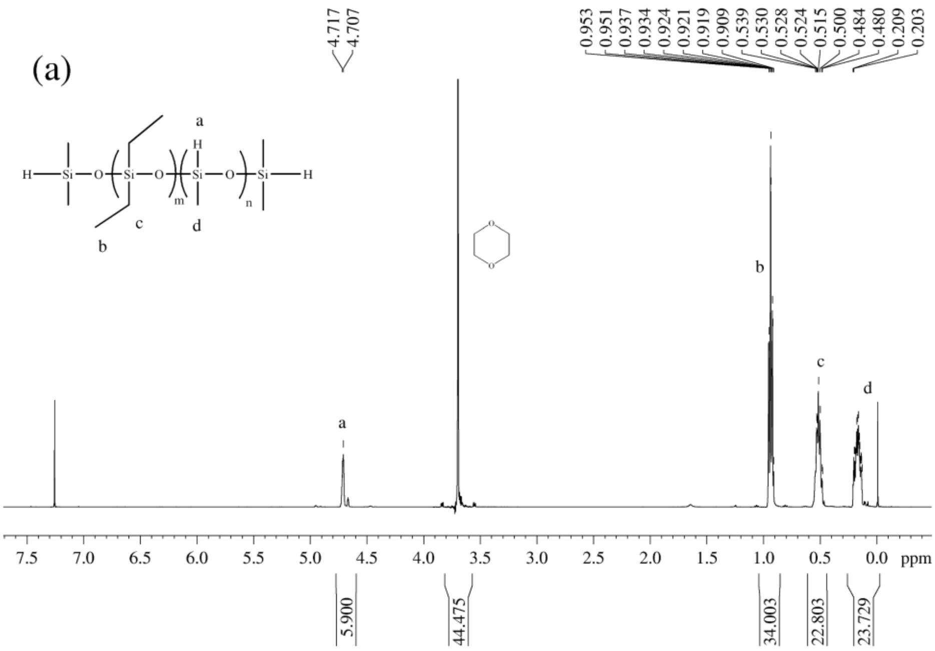
4.717
4.707
0.953
0.951
0.937
0.934
0.924
0.921
0.919
0.909
0.539
0.530
0.528
0.524
0.515
0.500
0.484
0.480
0.209
0.203
a
b
c
d
ppm
5.900
44.475
34.003
22.803
23.729

(b)

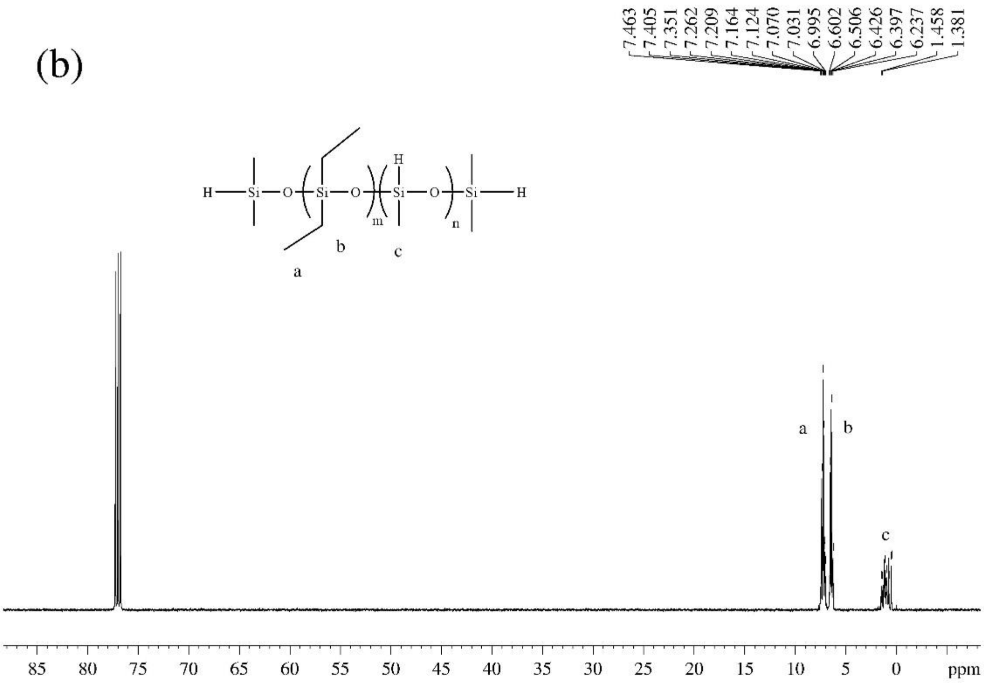
7.463
7.405
7.351
7.262
7.209
7.164
7.124
7.070
7.031
6.995
6.602
6.506
6.426
6.397
6.237
1.458
1.381
a
b
c
ppm

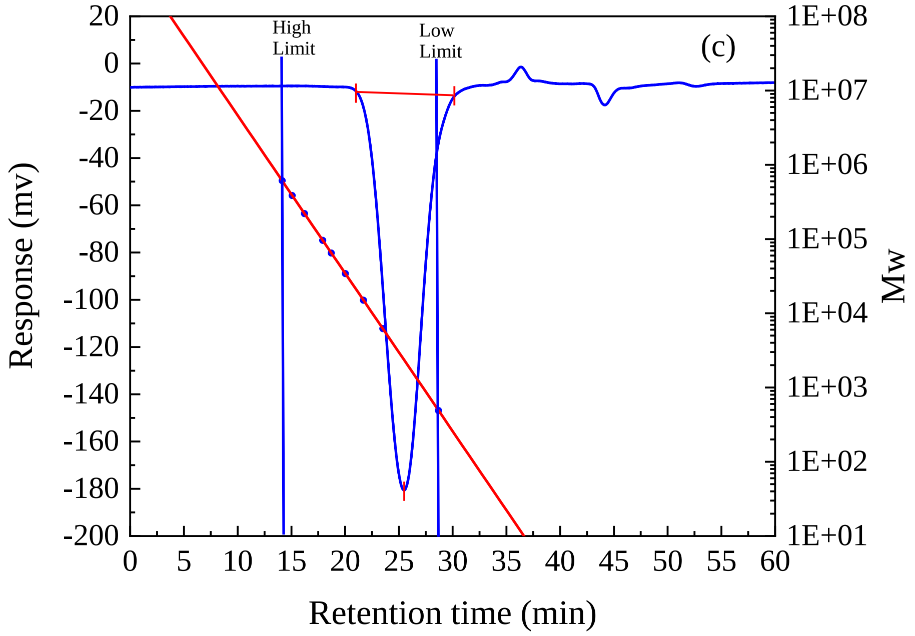

Figure S7. NMR spectra and GPC curve of α, ω-bisdimethylsiloxyl-terminated PMHS-*co*-PDES using $CDCl_3$ with trace amount of TMS as solvent (Entry 2E in Table 2. a. ^1H NMR spectrum of the mixture of 10.4 mg sample, 5.8 mg 1,4-dioxane and $CDCl_3$; b. ^{13}C NMR spectrum; c. GPC curve.)

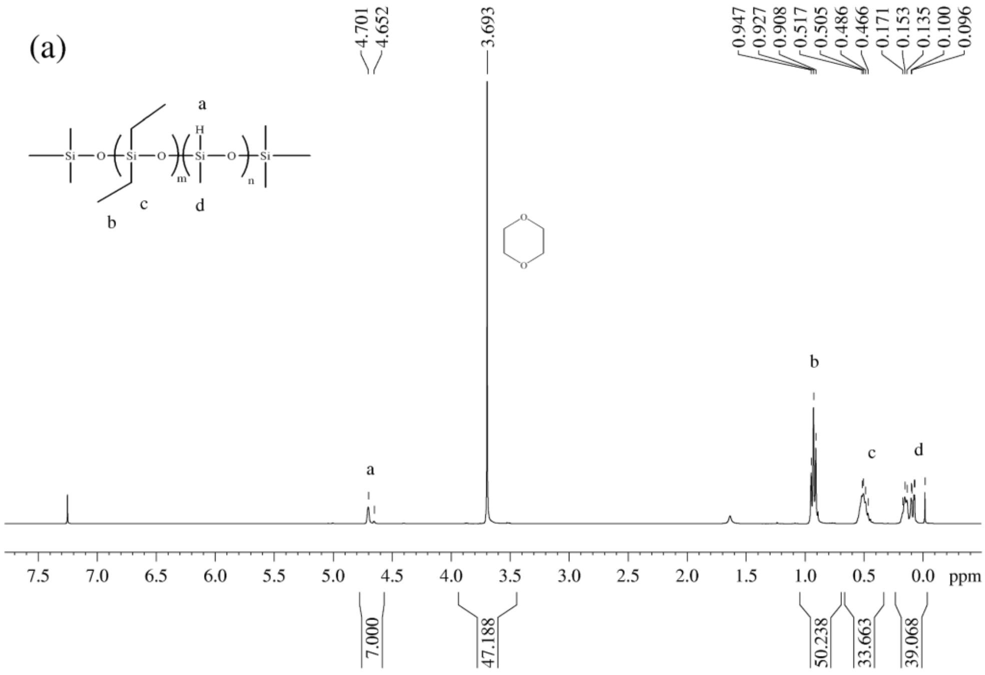

(b)

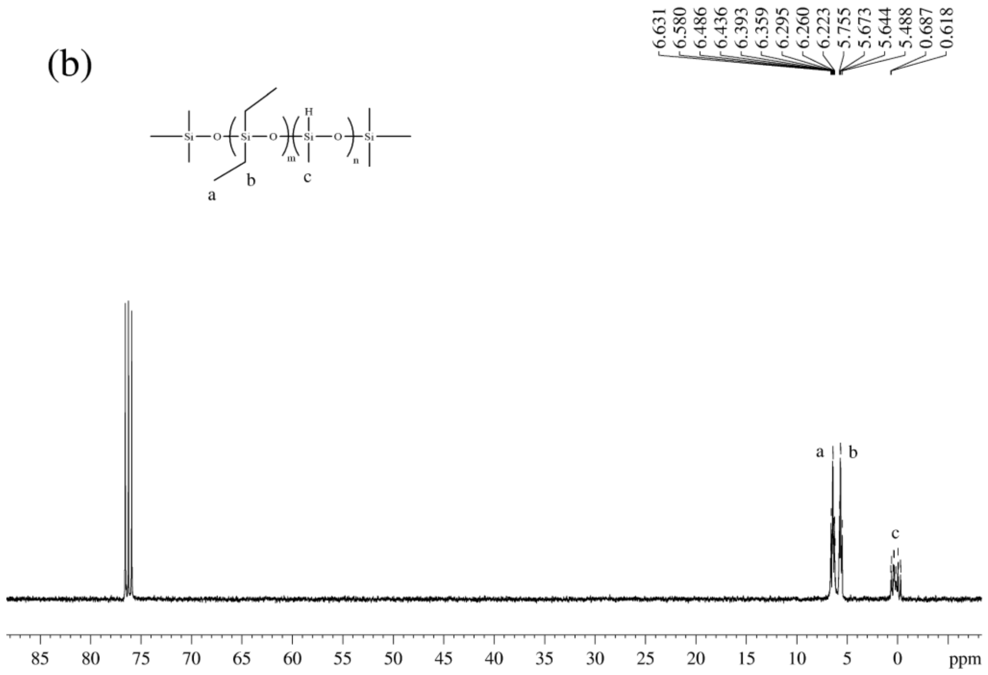

(c)

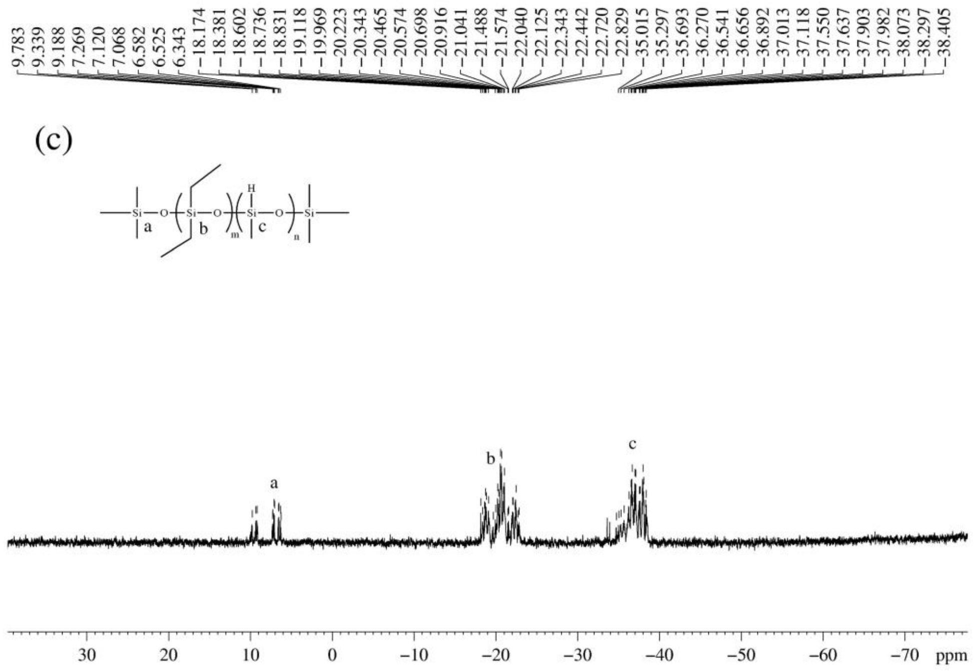

Figure S8. NMR spectra of α, ω-bistrimethylsiloxyl-terminated PMHS-*co*-PDES using $CDCl_3$ with trace amount of TMS as solvent (Entry 3A in Table 3. a. ^{1}H NMR spectrum of the mixture of 9.5 mg sample, 2.9 mg 1,4-dioxane and $CDCl_3$; b. ^{13}C NMR spectrum; c. ^{29}Si NMR spectrum.)

(a)

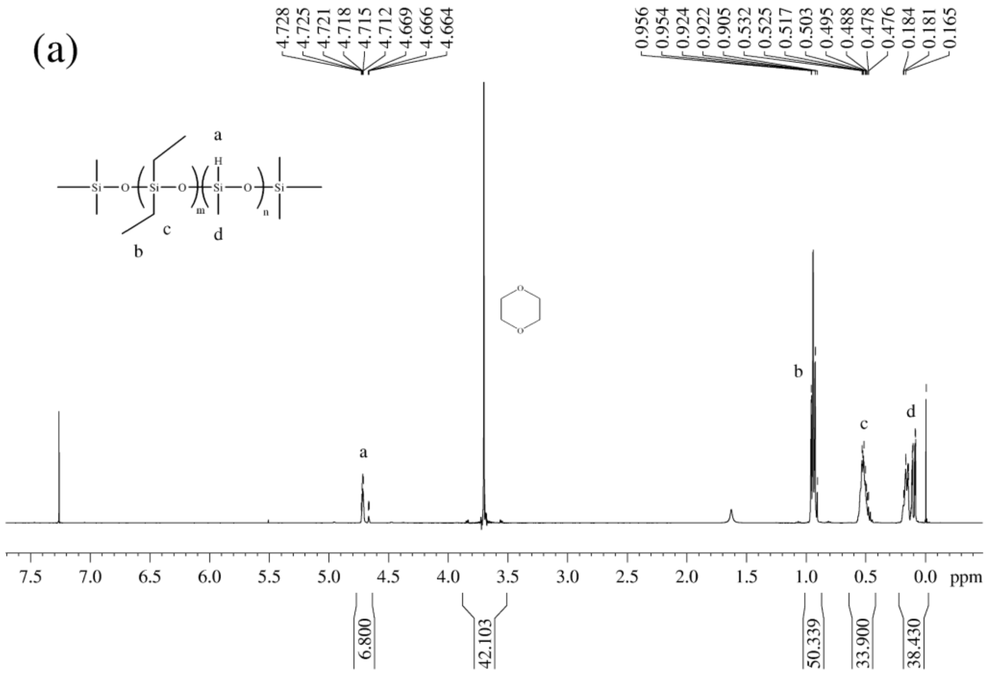
4.728
4.725
4.721
4.718
4.715
4.712
4.669
4.666
4.664
0.956
0.954
0.924
0.922
0.905
0.532
0.525
0.517
0.503
0.495
0.488
0.478
0.476
0.184
0.181
0.165
a
b
c
d
7.5 7.0 6.5 6.0 5.5 5.0 4.5 4.0 3.5 3.0 2.5 2.0 1.5 1.0 0.5 0.0 ppm
6.800
42.103
50.339
33.900
38.430

(b)

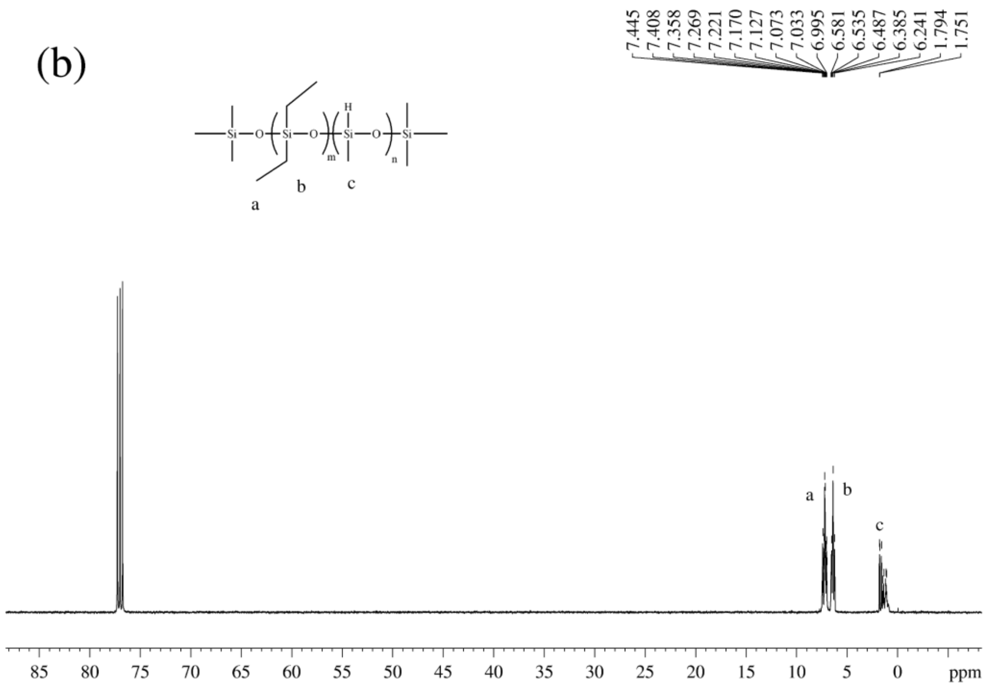
7.445
7.408
7.358
7.269
7.221
7.170
7.127
7.073
7.033
6.995
6.581
6.535
6.487
6.385
6.241
1.794
1.751
a
b
c
85 80 75 70 65 60 55 50 45 40 35 30 25 20 15 10 5 0 ppm

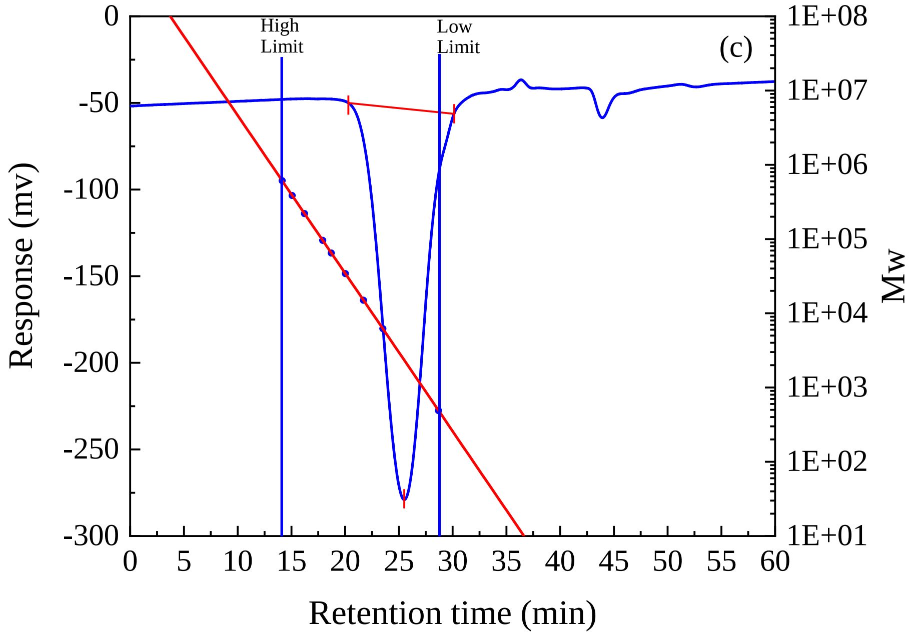

Figure S9. NMR spectra and GPC curve of *α*, *ω*-bistrimethylsiloxyl-terminated PMHS-*co*-PDES using $CDCl_3$ with trace amount of TMS as solvent (Entry 3B in Table 3. a. ^{1}H NMR spectrum of the mixture of 13.5 mg sample, 5.3 mg 1,4-dioxane and $CDCl_3$; b. ^{13}C NMR spectrum; c. GPC curve.)

(a)

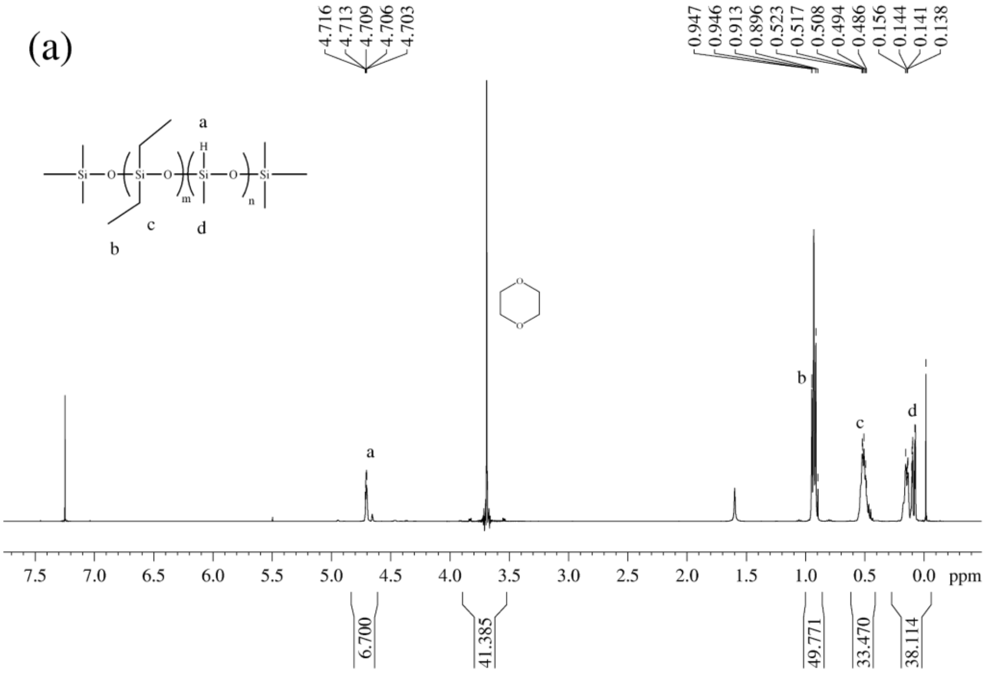
4.716
4.713
4.709
4.706
4.703
0.947
0.946
0.913
0.896
0.523
0.517
0.508
0.494
0.486
0.156
0.144
0.141
0.138
a
b
c
d
7.5 7.0 6.5 6.0 5.5 5.0 4.5 4.0 3.5 3.0 2.5 2.0 1.5 1.0 0.5 0.0 ppm
6.700
41.385
49.771
33.470
38.114

(b)

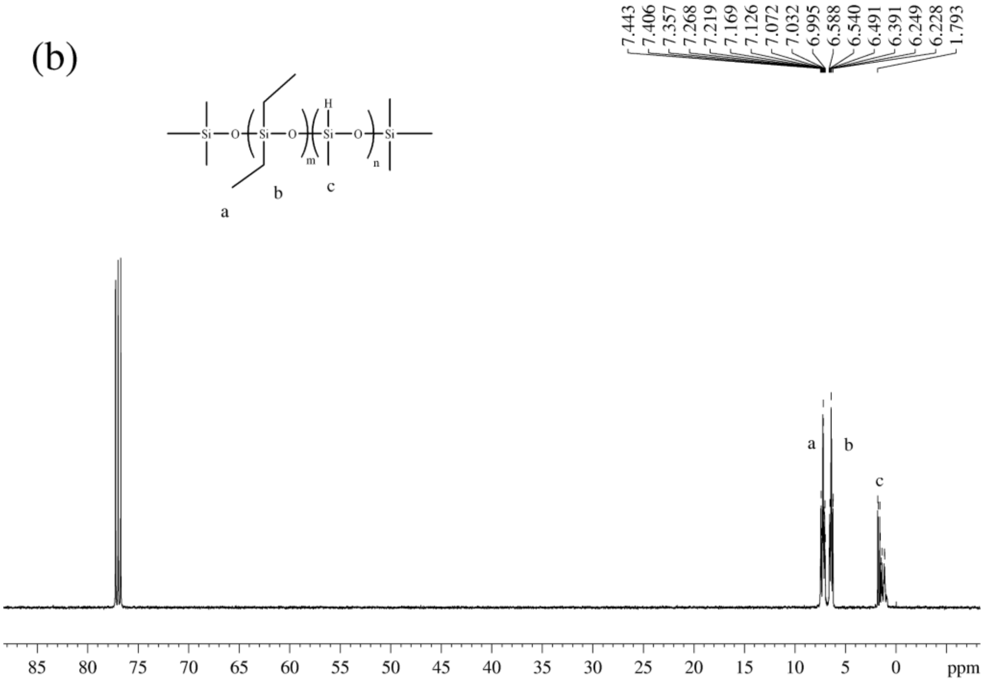
7.443
7.406
7.357
7.268
7.219
7.169
7.126
7.072
7.032
6.995
6.588
6.540
6.491
6.391
6.249
6.228
1.793
a
b
c
85 80 75 70 65 60 55 50 45 40 35 30 25 20 15 10 5 0 ppm

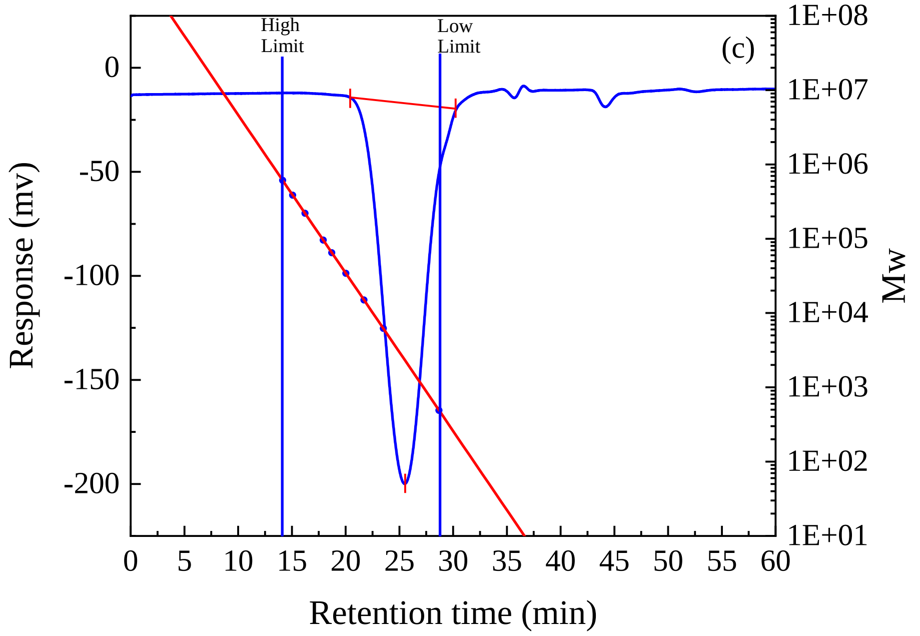

Figure S10. NMR spectra and GPC curve of *α*, *ω*-bistrimethylsiloxyl-terminated PMHS-*co*-PDES using $CDCl_3$ with trace amount of TMS as solvent (Entry 3C in Table 3. a. ^1H NMR spectrum of the mixture of 11.2 mg sample, 4.1 mg 1,4-dioxane and $CDCl_3$; b. ^{13}C NMR spectrum; c. GPC curve.)

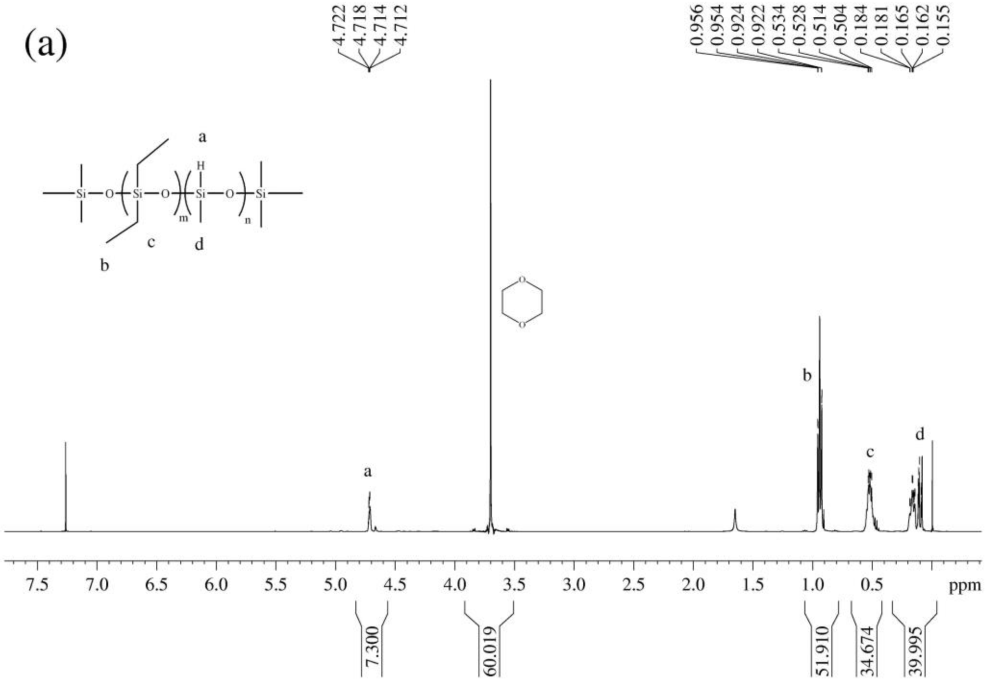

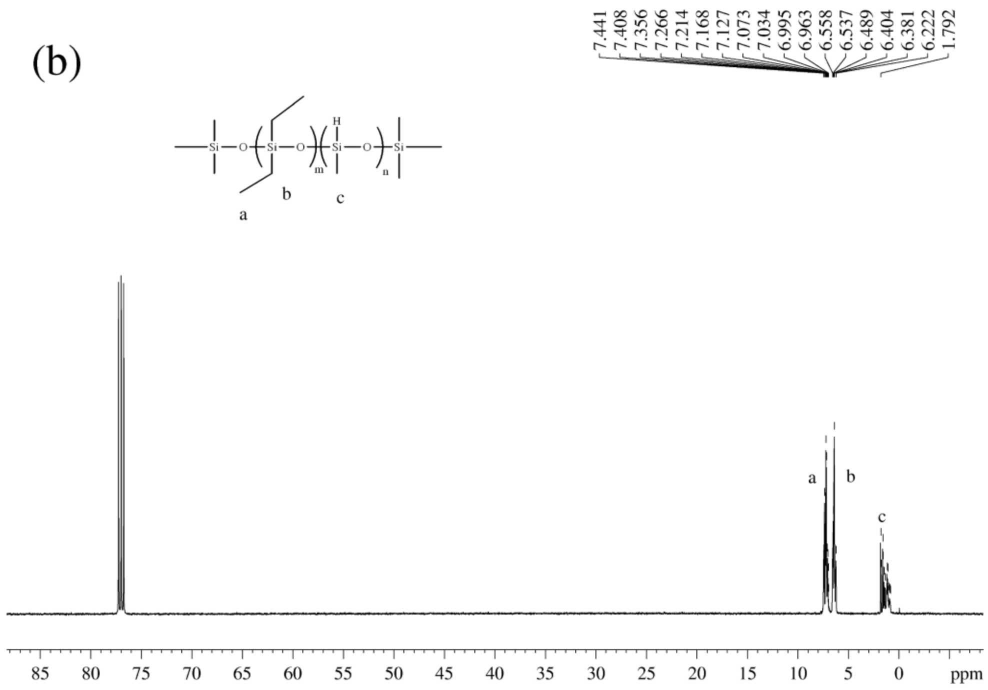

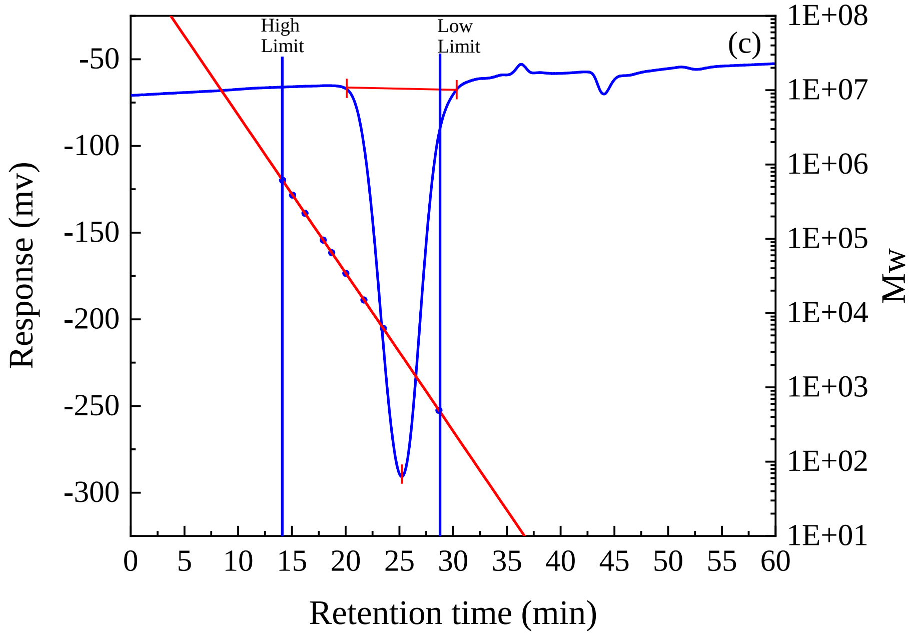

Figure S11. NMR spectra and GPC curve of *α*, *ω*-bistrimethylsiloxyl-terminated PMHS-*co*-PDES using $CDCl_3$ with trace amount of TMS as solvent (Entry 3D in Table 3. a. ^{1}H NMR spectrum of the mixture of 11.5 mg sample, 5.8 mg 1,4-dioxane and $CDCl_3$; b. ^{13}C NMR spectrum; c. GPC curve.)

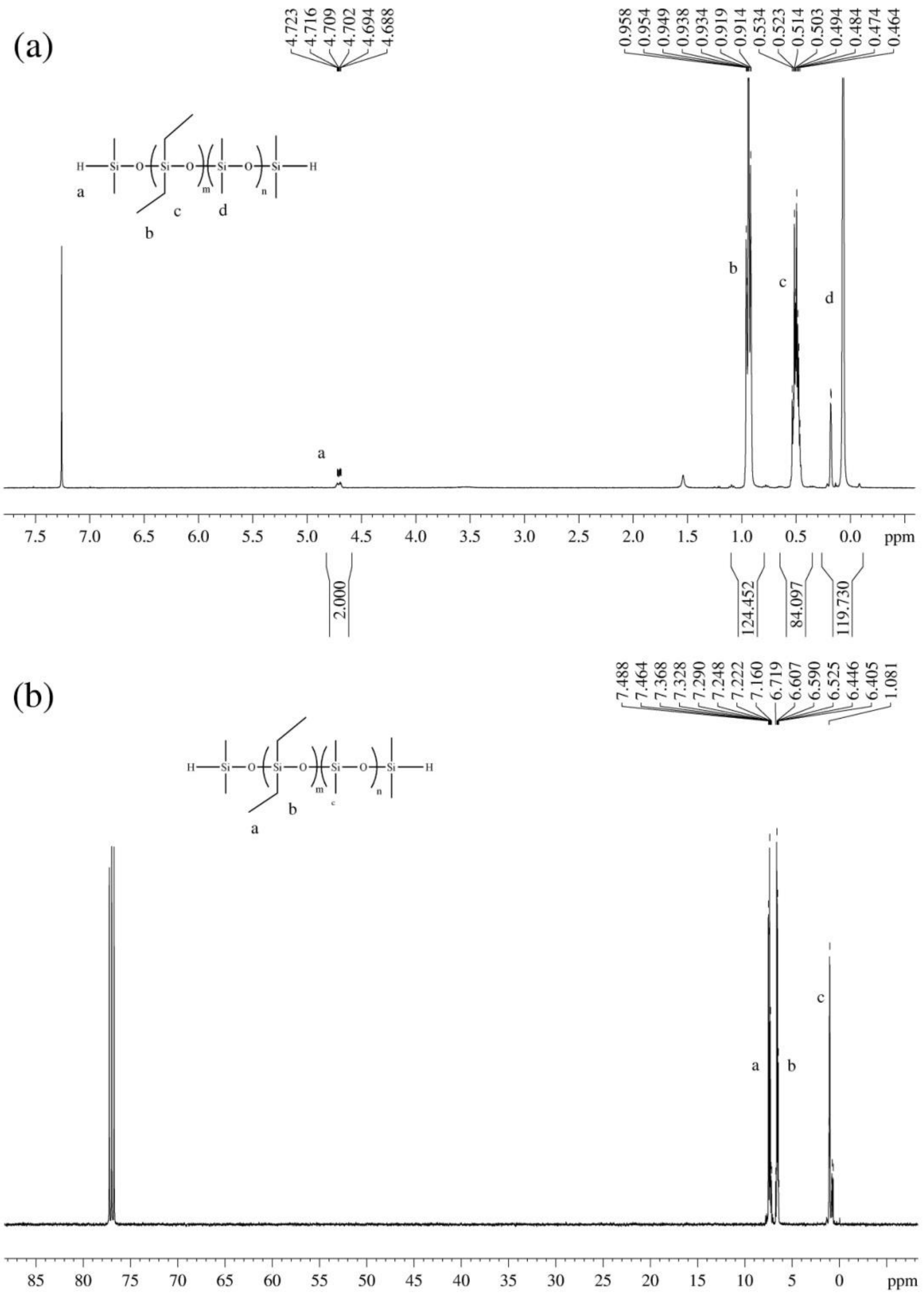
(a)
4.723
4.716
4.709
4.702
4.694
4.688
0.958
0.954
0.949
0.938
0.934
0.919
0.914
0.534
0.523
0.514
0.503
0.494
0.484
0.474
0.464
H
Si
O
m
n
a
b
c
d
7.5 7.0 6.5 6.0 5.5 5.0 4.5 4.0 3.5 3.0 2.5 2.0 1.5 1.0 0.5 0.0 ppm
2.000
124.452
84.097
119.730
(b)
7.488
7.464
7.368
7.328
7.290
7.248
7.222
7.160
6.719
6.607
6.590
6.525
6.446
6.405
1.081
85 80 75 70 65 60 55 50 45 40 35 30 25 20 15 10 5 0 ppm

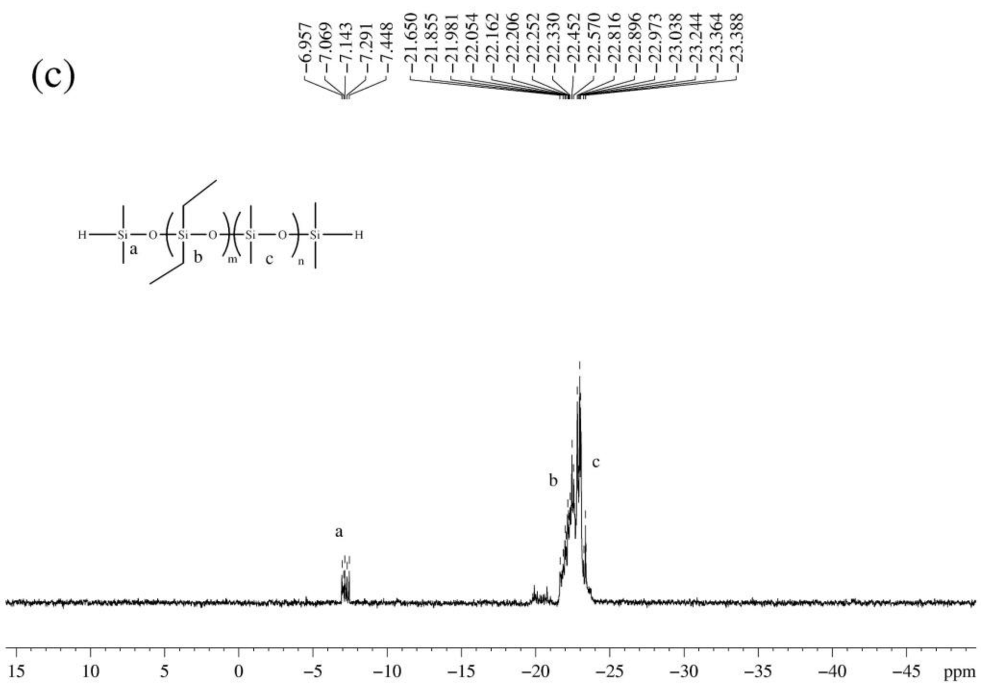

Figure S12. NMR spectra of α, ω-bisdimethylsiloxyl-terminated PDMS-*co*-PDES using $CDCl_3$ with trace amount of TMS as solvent (Entry 4A in Table 4. a. ^{1}H NMR spectrum; b. ^{13}C NMR spectrum; c. ^{29}Si NMR spectrum.)

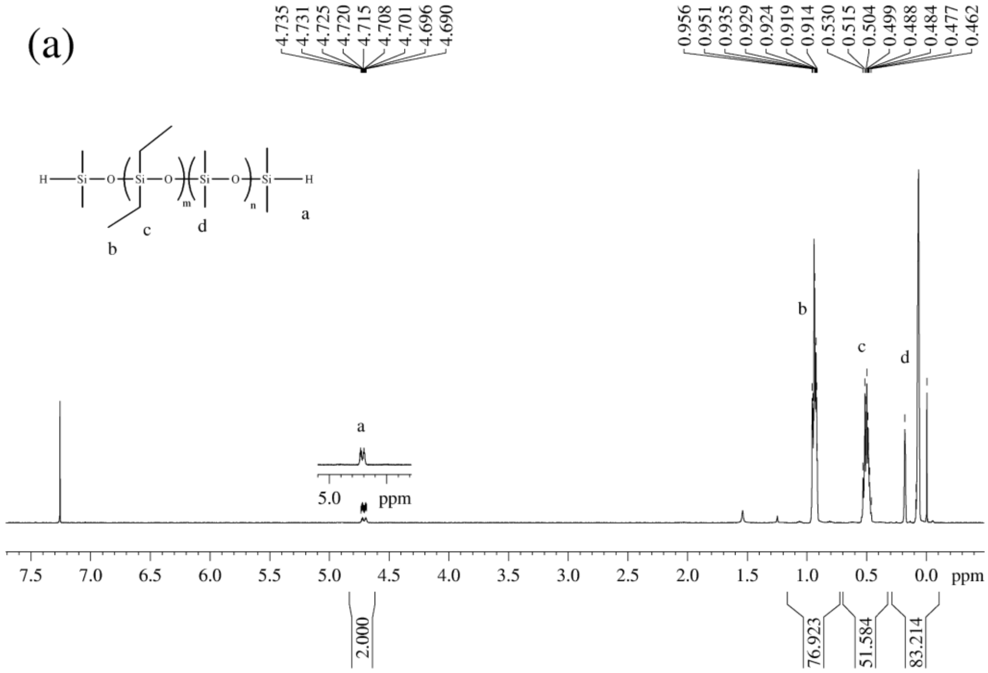

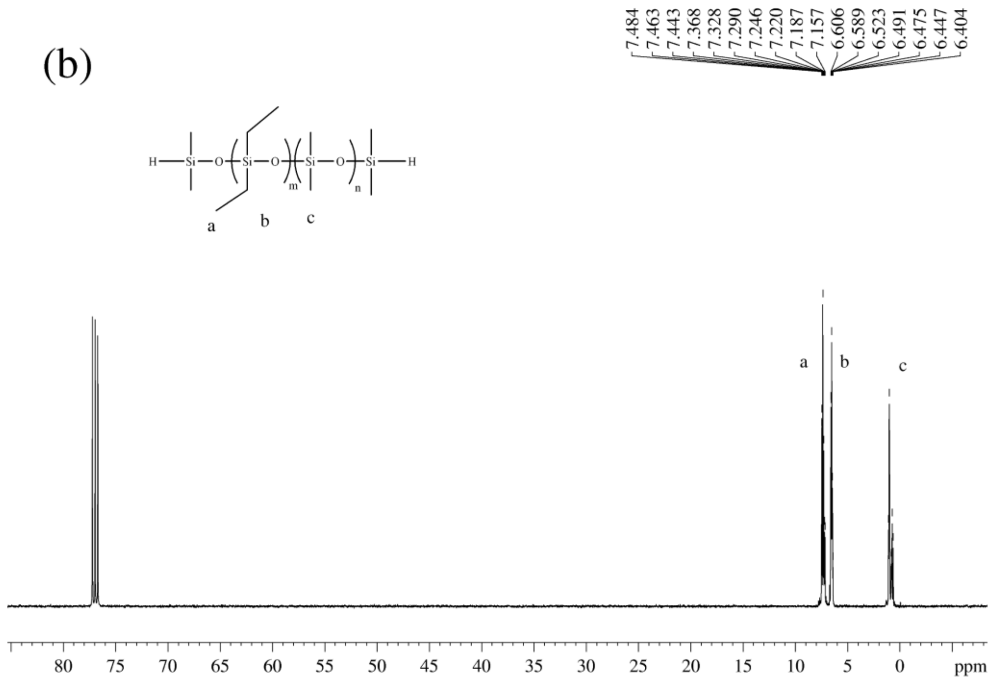

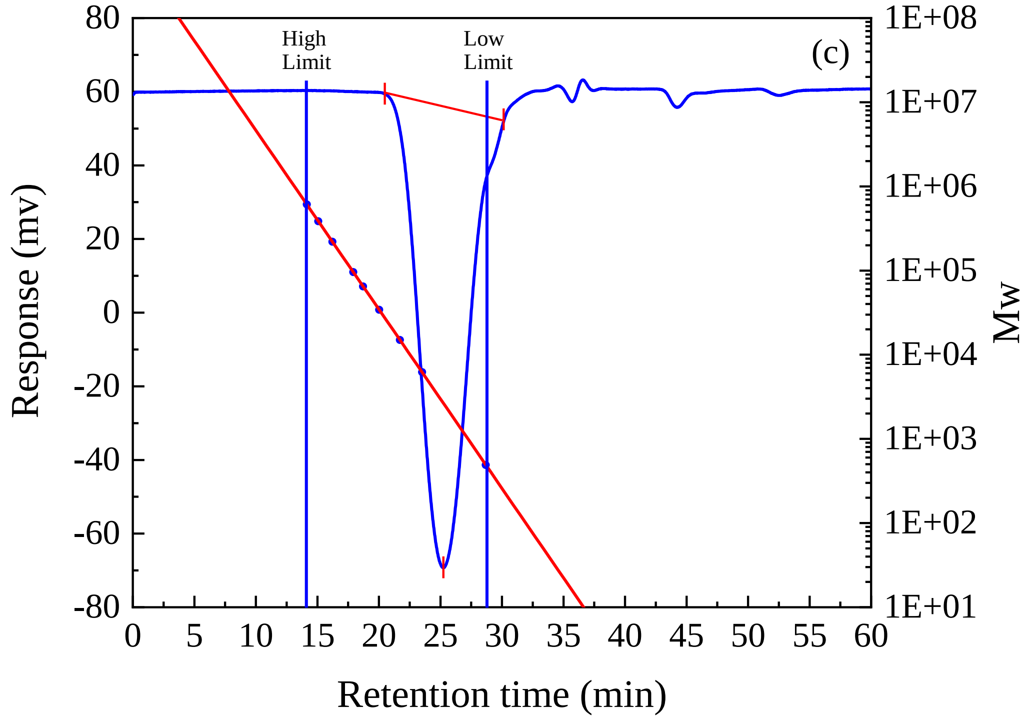

Figure S13. NMR spectra and GPC curve of *α*, *ω*-bisdimethylsiloxyl-terminated PDMS-*co*-PDES using $CDCl_3$ with trace amount of TMS as solvent (Entry 4B in Table 4. a. ^1H NMR spectrum; b. ^{13}C NMR spectrum; c. GPC curve.)

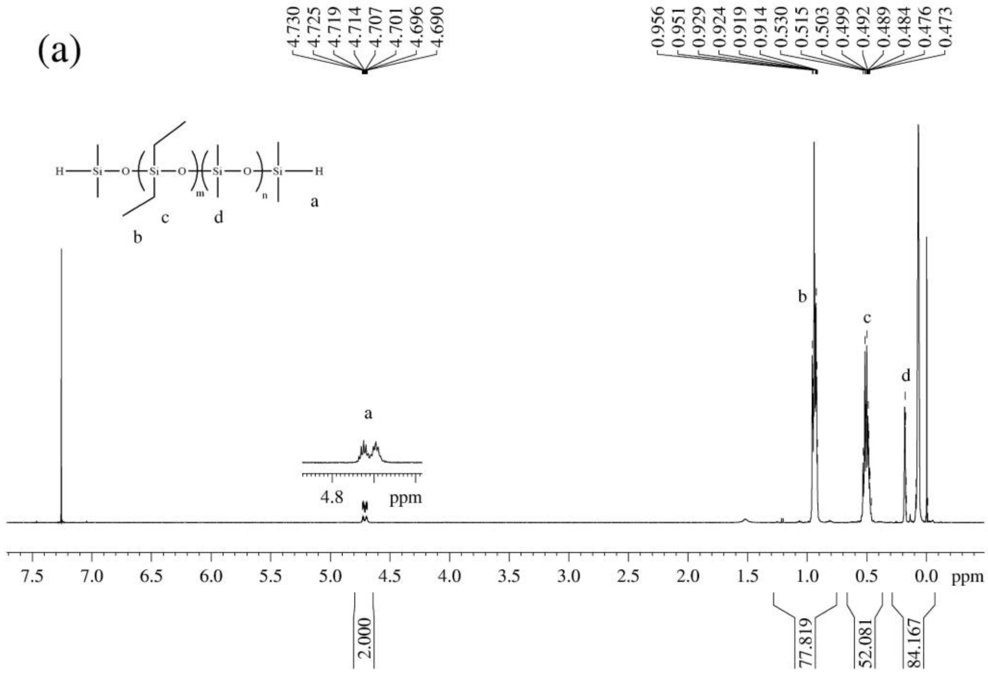
(a)
4.730
4.725
4.719
4.714
4.707
4.701
4.696
4.690
0.956
0.951
0.929
0.924
0.919
0.914
0.530
0.515
0.503
0.499
0.492
0.489
0.484
0.476
0.473
H
Si
O
Si
O
m
Si
O
n
Si
H
a
b
c
d
b
c
d
a
4.8
ppm
7.5
7.0
6.5
6.0
5.5
5.0
4.5
4.0
3.5
3.0
2.5
2.0
1.5
1.0
0.5
0.0
ppm
2.000
77.819
52.081
84.167

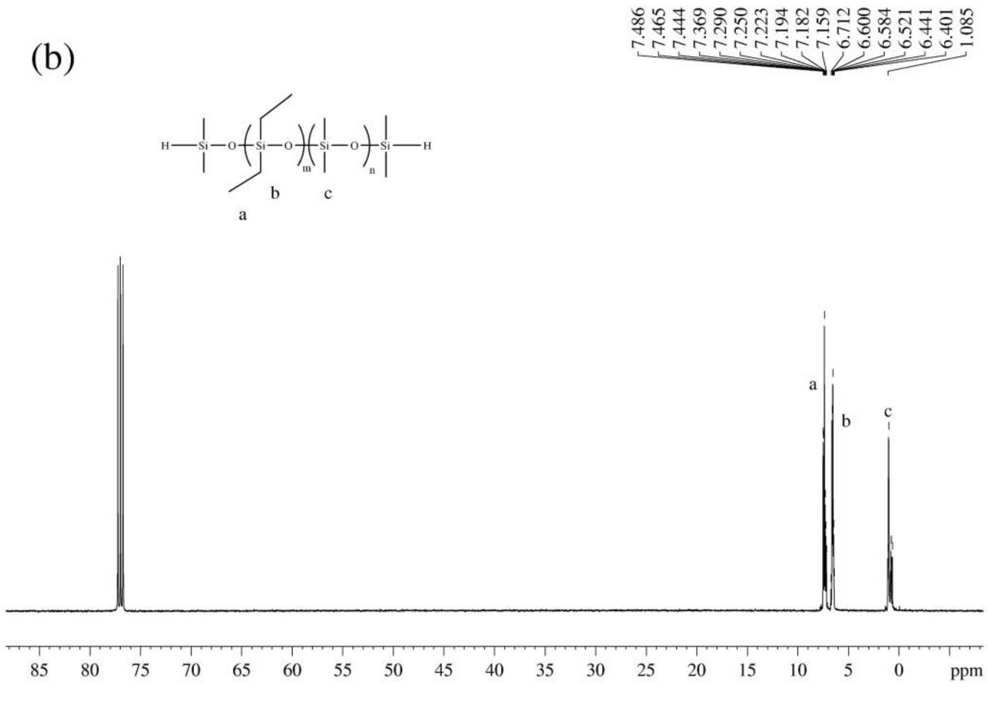
(b)
7.486
7.465
7.444
7.369
7.290
7.250
7.223
7.194
7.182
7.159
6.712
6.600
6.584
6.521
6.441
6.401
1.085
H
Si
O
Si
O
m
Si
O
n
Si
H
a
b
c
a
b
c
85
80
75
70
65
60
55
50
45
40
35
30
25
20
15
10
5
0
ppm

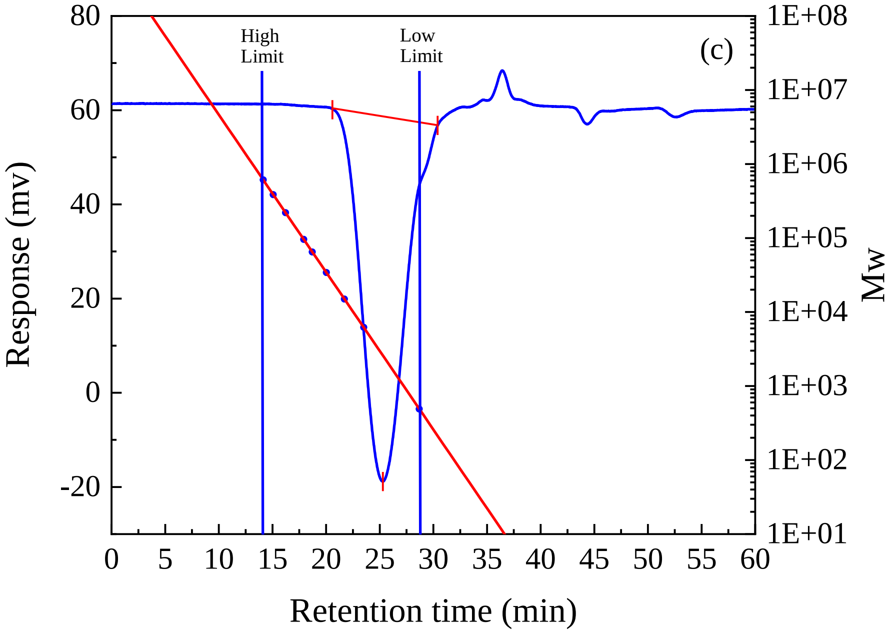

Figure S14. NMR spectra and GPC curve of *α*, *ω*-bisdimethylsiloxyl-terminated PDMS-*co*-PDES using $CDCl_3$ with trace amount of TMS as solvent (Entry 4C in Table 4. a. ^{1}H NMR spectrum; b. ^{13}C NMR spectrum; c. GPC curve.)

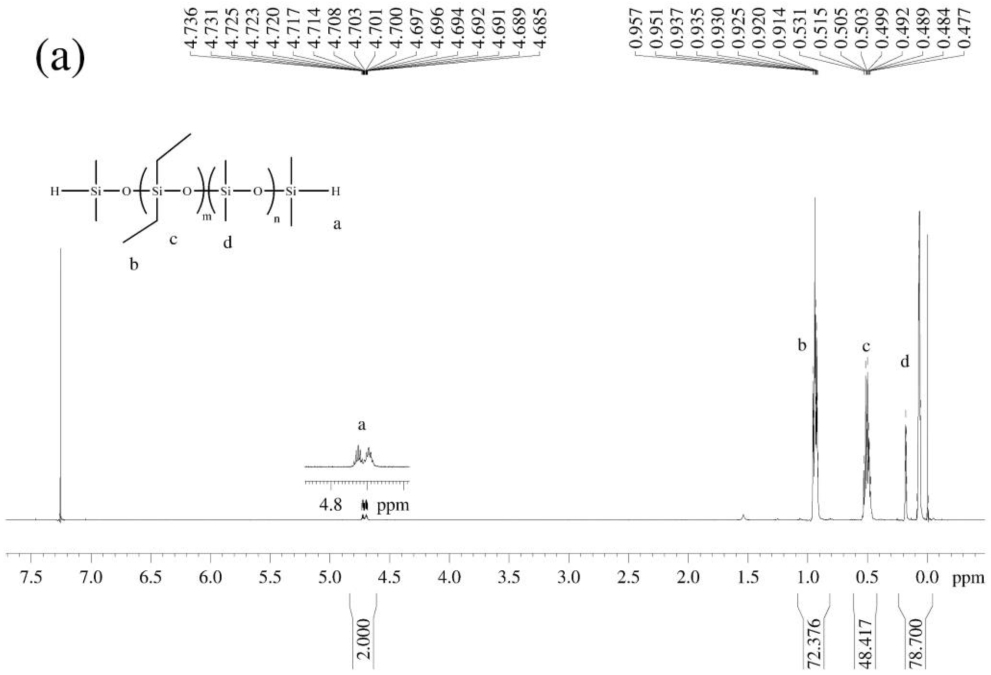

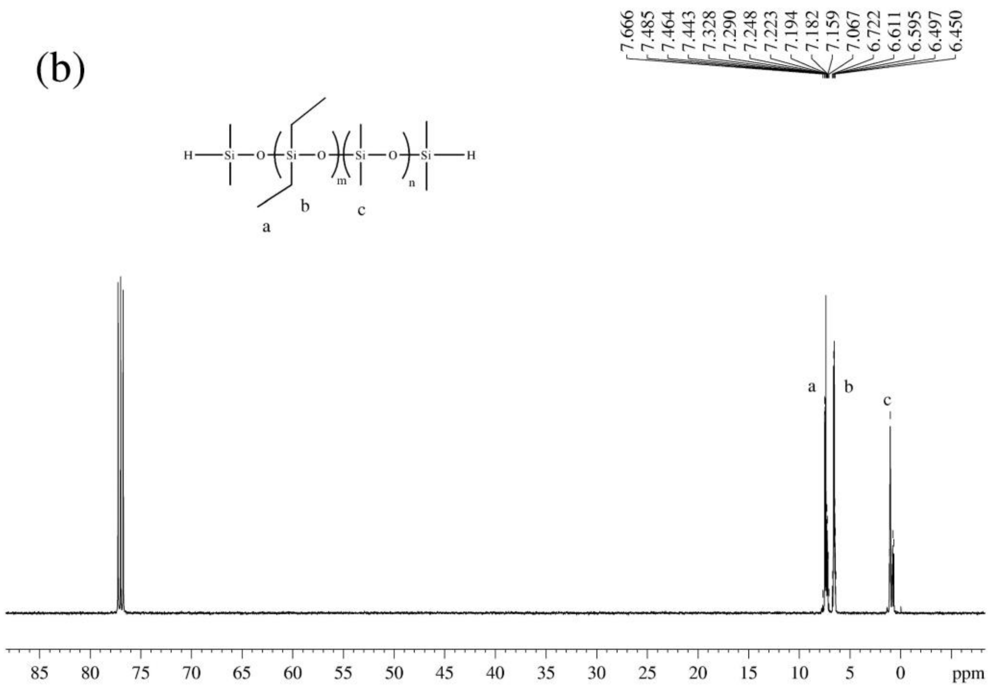

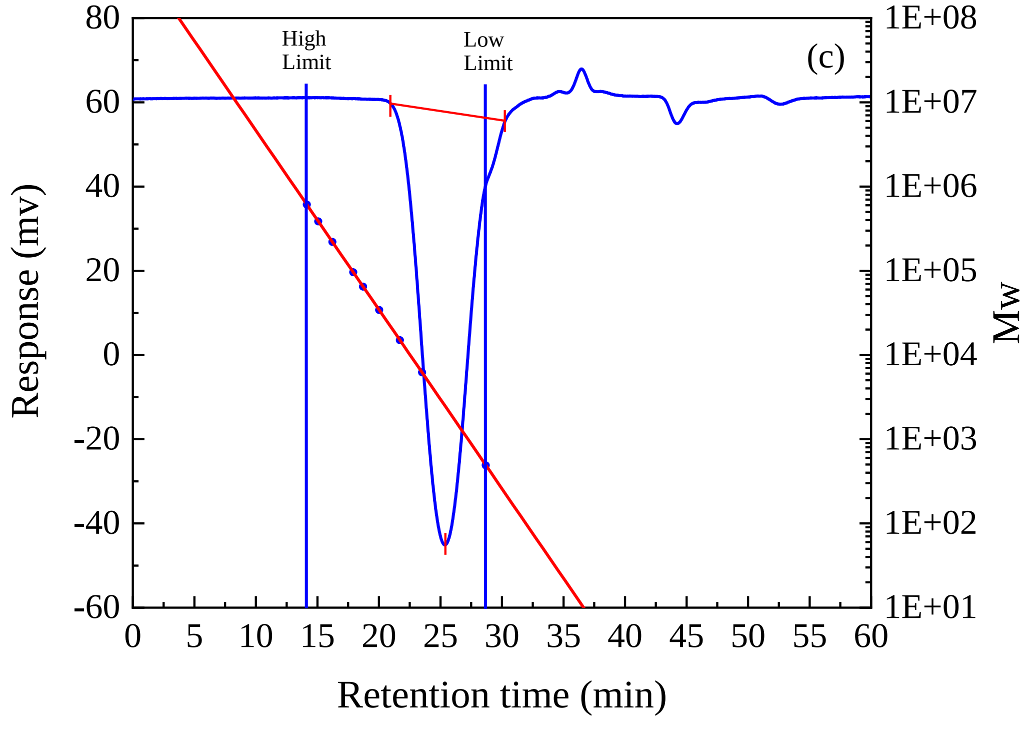

Figure S15. NMR spectra and GPC curve of *α*, *ω*-bisdimethylsiloxyl-terminated PDMS-*co*-PDES using $CDCl_3$ with trace amount of TMS as solvent (Entry 4D in Table 4. a. ^{1}H NMR spectrum; b. ^{13}C NMR spectrum; c. GPC curve.)

(a)

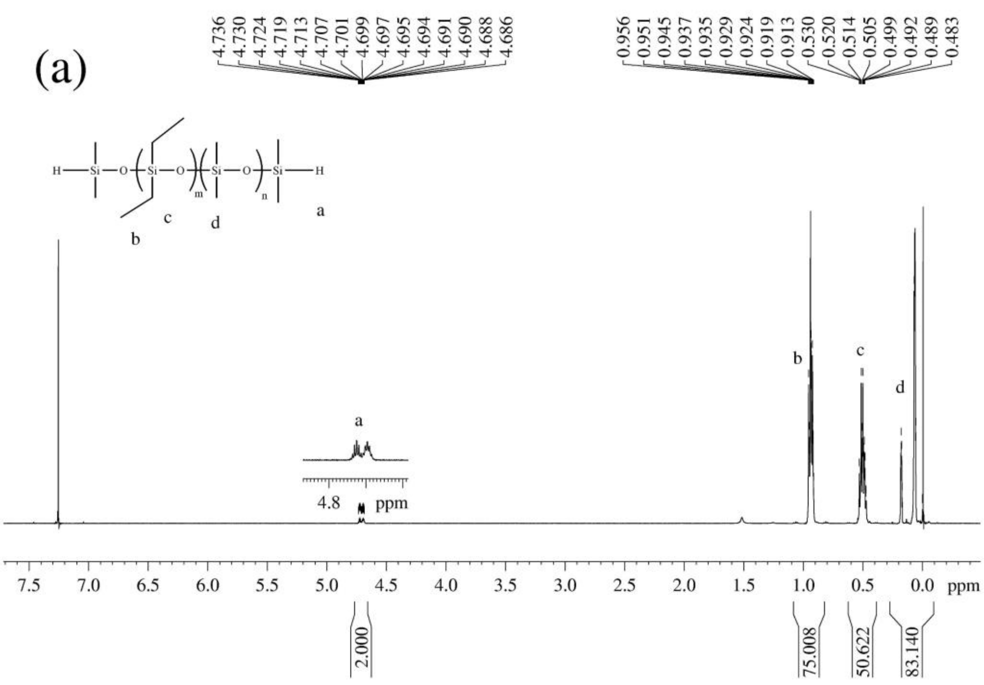

4.736
4.730
4.724
4.719
4.713
4.707
4.701
4.699
4.697
4.695
4.694
4.691
4.690
4.688
4.686
0.956
0.951
0.945
0.937
0.935
0.929
0.924
0.919
0.913
0.530
0.520
0.514
0.505
0.499
0.492
0.489
0.483
H
Si
O
m
n
a
b
c
d
4.8
ppm
7.5
7.0
6.5
6.0
5.5
5.0
4.5
4.0
3.5
3.0
2.5
2.0
1.5
1.0
0.5
0.0
2.000
75.008
50.622
83.140

(b)

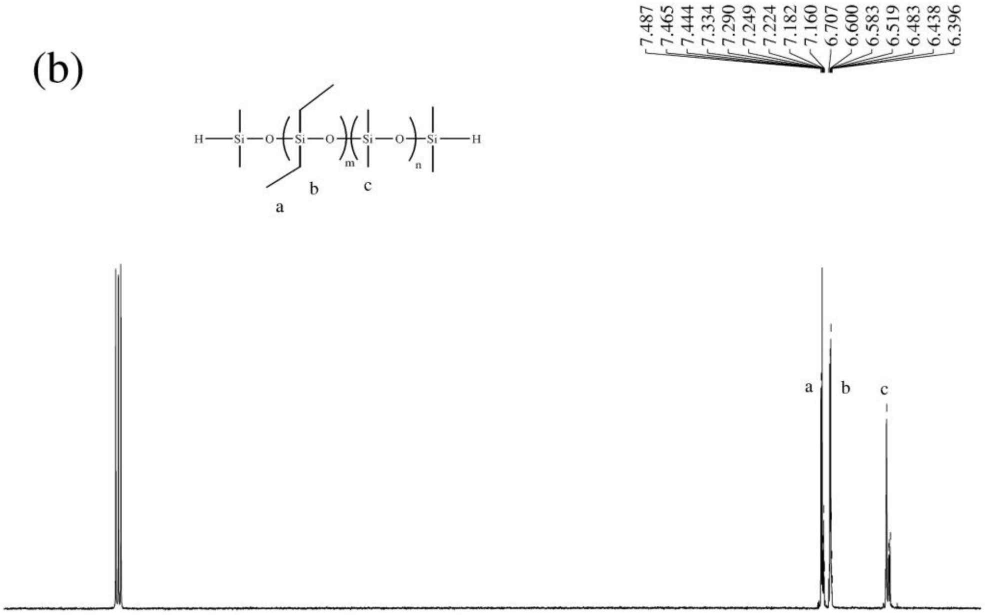

7.487
7.465
7.444
7.334
7.290
7.249
7.224
7.182
7.160
6.707
6.600
6.583
6.519
6.483
6.438
6.396
H
Si
O
m
n
a
b
c
85
80
75
70
65
60
55
50
45
40
35
30
25
20
15
10
5
0
ppm

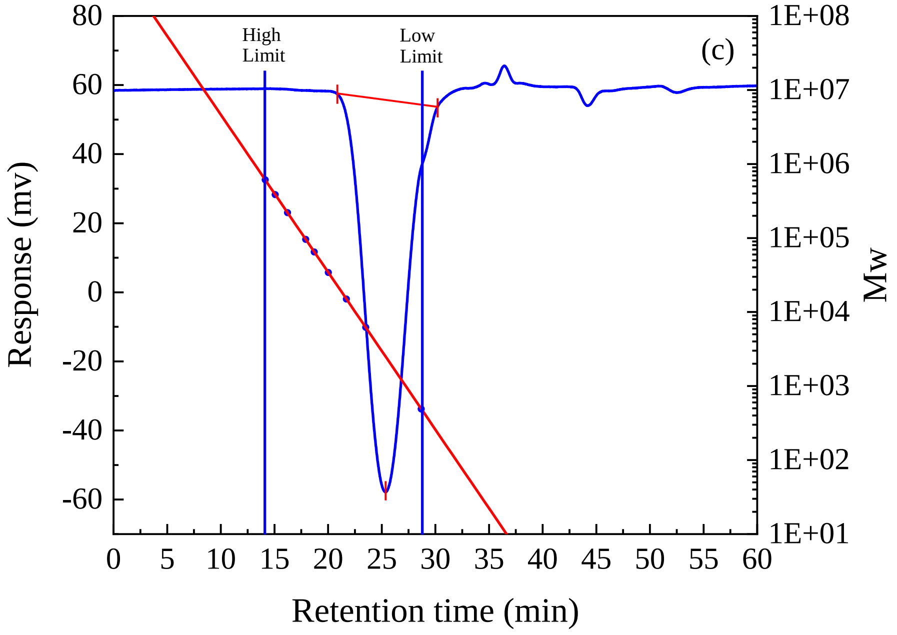

Figure S16. NMR spectra and GPC curve of *α*, *ω*-bisdimethylsiloxyl-terminated PDMS-*co*-PDES using $CDCl_3$ with trace amount of TMS as solvent (Entry 4E in Table 4. a. ^1H NMR spectrum; b. ^{13}C NMR spectrum; c. GPC curve.)

Part II. Calculation of molecular weight (M_{NMR}) and degree of polymerization for PDES oligomers or copolymers from ^{1}H NMR spectrum

S2.1 α, ω-bisdimethylsiloxyl-terminated PDES oligomers (PDES-H)

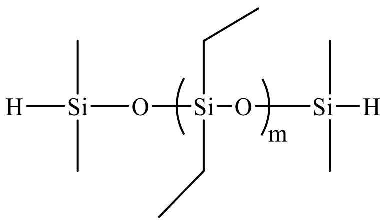

Chart S1. Structure formula of α, ω-bisdimethylsiloxyl-terminated PDES oligomers

Taking Entry 1C in Table 1 as an example. Its ^{1}H NMR spectrum is shown in Figure S1(a) and its chemical structure is shown in Chart S1. If defining the area integration of Si-H as 2, then the area integration of methyl group in Et_2SiO segments is 68.273, the area integration of methylene group in Et_2SiO segments is 46.013, and the area integration of methyl group in end-capping group ($Me_2HSiO_{1/2}$-) is 12.132. From the structure formula of this polymer, the following two equations (S1 and S2) could be deduced.

$$4m = 46.013 \quad \text{(S1)}$$

$$6m = 68.273 \quad \text{(S2)}$$

When solving these equations, the value of m could be obtained with a result of $m \approx 11$. The molecular weight of this polymer could be calculated as:

$$M_{NMR}=134+11\times102=1256 \text{ g/mol} \quad \text{(S3)}$$

The designed molecular weight of this polymer is 1052 g/mol.

The degree of polymerization (m) and molecular weight (M_{NMR}) of other PDES-H fluids were calculated according to the above method, and the results are listed in columns 8 and 10 of Table 1.

S2.2 *α*, *ω*-bisdimethylvinylsiloxyl-terminated PDES oligomers (PDES-Vi)

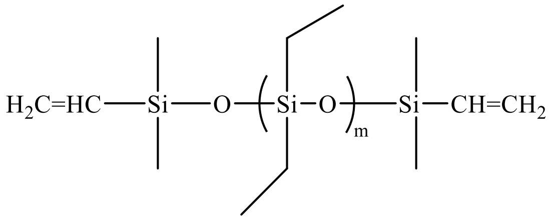

Chart S2. Structure formula of *α*, *ω*-bisdimethylvinylsiloxyl-terminated PDES oligomers

According to the structural formula of *α*, *ω*-bisdimethylvinylsiloxyl-terminated PDES oligomers (PDES-Vi) shown in Chart S2 and Figure S2(a), if the area integration of Si-CH=CH_2 is defined as 6, the area integration of the peak of Si-CH_3 is close to 12. Because the repeating unit of this oligomer is Et_2SiO, the ratio of protons in the CH_2 group and CH_3 group is 2: 3. According to the integrated area of these two kinds of protons, it can be calculated that m=13. Therefore, the molecular weight (M_{NMR}) of this oligomer is 1512 g/mol, and the vinyl content is 3.57%.

S2.3 *α*, *ω*-bisdimethylsiloxyl-terminated PMHS-*co*-PDES

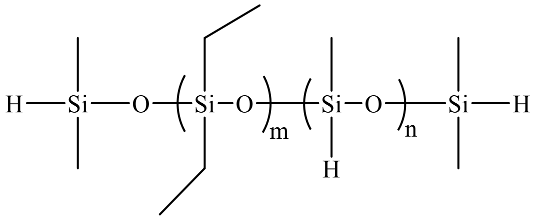

Chart S3. Structure formula of *α*, *ω*-bisdimethylsiloxyl-terminated PMHS-*co*-PDES

Taking Entry 2A in Table 2 as an example, its chemical structure is shown in Chart S3. The calculation of the degree of polymerization of Et_2SiO segments (m) and that of MeHSiO segments (n) is illustratively shown below.

According to the structural formula of *α*, *ω*-bisdimethylsiloxyl-terminated PMHS-*co*-PDES, when 2.8 mg of 1,4-dioxane was used as an external standard, blended with 9.7 mg of the copolymer in $CDCl_3$ solvent, its ^{1}H NMR spectrum is shown in Figure S3(a). Suppose the integrated area of

Si-H is defined as 5. In that case, the integrated area of the H proton peak in 1,4-dioxane is 21.8, and the contents of hydrogen and methyl groups in this copolymer can be calculated as shown in equation (S4) and equation (S5), respectively.

$$H\% = \frac{S_H \times m_E \times 8}{S_E \times m_a \times 88.11} \times 100\% \qquad \text{(S4)}$$

$$CH_3\% = \frac{S_{Me} \times m_E \times 8}{S_E \times m_a \times 88.11} \times 100\% \qquad \text{(S5)}$$

In equations S4 and S5, S_H is the area integration of Si-H in ^{1}H NMR spectra located at about δ=4.7 ppm, m_E is the mass weight of the 1,4-dioxane, S_E is the area integration of H atoms resulting from 1,4-dioxane located at about δ=3.7 ppm, S_{Me} is the area integration of protons resulting from Si-CH_3 located at about δ=0.16 ppm, m_a is the mass weight of the sample blended with the 1,4-dioxane, the number 88.11 is the molar weight of 1,4-dioxane. The number 8 is the number of protons in 1 mole of 1,4-dioxane. When each parameter is put into equations S4 and S5, the contents of H and methyl are calculated to be 0.60% and 2.52%, respectively. According to the structural formula of the copolymer and equations (S4) and (S5), the content of the hydrogen and methyl groups in the copolymer can be expressed in equations (S6) and (S7), respectively.

$$H\% = \frac{n+2}{102m+60n+134} \times 100\% = 0.60\% \qquad \text{(S6)}$$

$$CH_3\% = \frac{3n+12}{102m+60n+134} \times 100\% = 2.52\% \qquad \text{(S7)}$$

When solving equations (S6) and (S7), values of m=5.1 and n=3.0 could be obtained. The molecular weight of the copolymer can be calculated as M_{NMR}=834 g/mol.

From Figures S4 to S7, the polymerization degree m of the Et_2SiO segments and n of MeHSiO segments in other α, ω-bisdimethylsiloxyl-terminated PMHS-*co*-PDES could be obtained according to the same method. The values of the degree of polymerization (m) of Et_2SiO segments and that of

MeHSiO segment (n) and the values of M_{NMR} of α, ω-bisdimethylsiloxyl-terminated PMHS-*co*-PDES are presented in columns 8 to 10 of Table 2, respectively.

S2.4 α, ω-bistrimethylsiloxyl-terminated PMHS-*co*-PDES

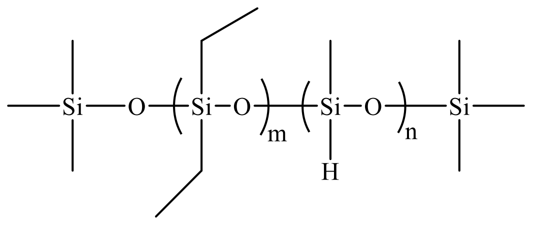

Chart S4. Structure formula of α, ω-bistrimethylsiloxyl-terminated PMHS-*co*-PDES

The structure of α, ω-bistrimethylsiloxyl-terminated PMHS-*co*-PDES is shown in Chart S4. There are repeated MeHSiO and Et_2SiO segments in the copolymer molecule, and Si-H exists only in the side chains of the copolymer. Using 1,4-dioxane as an external standard, the degree of polymerization of each repeating unit can be calculated. Taking Entry 3A in Table 3 as an example, the polymerization degree (m) of Et_2SiO segments and the polymerization degree (n) of MeHSiO segments in the copolymer are calculated as follows.

When 2.9 mg of 1,4-dioxane was used as an external standard, blended with 9.5 mg of the copolymer in $CDCl_3$ solvent, its ^1H NMR spectrum is shown in Figure S8(a). According to this copolymer's structural formula, if the integrated area of Si-H is defined as 7, the integrated area of the H proton peak in 1,4-dioxane is 47.2. Therefore, the hydrogen content and methyl group content of this copolymer can be calculated by equations S4 and S5, and the results are 0.41% and 2.29%, respectively.

According to the structural formula of the copolymer and equations (S4) and (S5), the content of the hydrogen and methyl groups in the copolymer can be expressed in equations (S8) and (S9), respectively.

$$H\% = \frac{n+2}{102m+60n+134} \times 100\% = 0.41\% \quad \text{(S8)}$$

$$CH_3\% = \frac{3n+12}{102m+60n+134} \times 100\% = 2.29\% \quad \text{(S9)}$$

When solving equations (S8) and (S9), values of m=11.0 and n=7.0 could be obtained. The molecular weight of the copolymer can be calculated as M_{NMR}=1704 g/mol.

From Figures S9 to S11, the polymerization degree m of the Et_2SiO segments and n of MeHSiO segments in other *α*, *ω*-bistrimethylsiloxyl-terminated PMHS-*co*-PDES samples could be obtained according to the same method. The values of the degree of polymerization (m) of Et_2SiO segments and that of MeHSiO segment (n) and the values of M_{NMR} of *α*, *ω*-bistrimethylsiloxyl-terminated PMHS-*co*-PDES samples are presented in columns 7 to 9 of Table 3, respectively.

S2.5 *α*, *ω*-bisdimethylsiloxyl-terminated PDMS-*co*-PDES

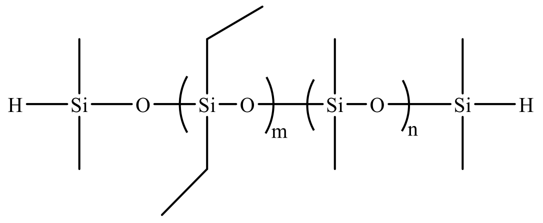

Chart S5. Structure formula of *α*, *ω*-bisdimethylsiloxyl-terminated PDMS-*co*-PDES

The structure of *α*, *ω*-bisdimethylsiloxyl-terminated PDMS-*co*-PDES is shown in Chart S5. Taking Entry 4A in Table 4 as an example, its ^{1}H NMR spectrum is shown in Figure S12(a). If the integrated area of Si-H is defined as 2, the integrated area of Si-CH_3 is 120. Because the molar ratio of the protons in the methylene group to the protons in the methyl group in the repeated Et_2SiO segments is 2: 3, the degree of polymerization (m) of Et_2SiO segments and the degree of polymerization (n) of Me_2SiO segments can be calculated as m=20 and n=18 on the basis of the integrated area of these two kinds of protons in Figure S12(a). The molecular weight M_{NMR} of this copolymer could be further calculated as 3506 g/mol. The values of the degree of polymerization

(m) of Et_2SiO segments and that of Me_2SiO segments (n) and the values of M_{NMR} of α, ω-bisdimethylsiloxyl-terminated PDMS-*co*-PDES are presented in columns 8 to 10 of Table 4, respectively.
